# Inhibitory and stimulatory micropeptides preferentially bind to different conformations of the cardiac calcium pump

**DOI:** 10.1016/j.jbc.2022.102060

**Published:** 2022-05-20

**Authors:** Sean R. Cleary, Xuan Fang, Ellen E. Cho, Marsha P. Pribadi, Jaroslava Seflova, Jordan R. Beach, Peter M. Kekenes-Huskey, Seth L. Robia

**Affiliations:** Department of Cell and Molecular Physiology, Loyola University Chicago, Maywood, Illinois, USA

**Keywords:** binding kinetics, Bowditch effect, calcium signaling, calcium transporter, conformational selection, force–frequency relationship, membrane protein–protein interactions, micropeptides, physiological model, transport ATPase, DWORF, dwarf open reading frame, ER, endoplasmic reticulum, PLB, phospholamban, SERCA, sarco/endoplasmic reticulum Ca^2+^-ATPase, SR, sarcoplasmic reticulum, TG, thapsigargin

## Abstract

The ATP-dependent ion pump sarco/endoplasmic reticulum Ca^2+^-ATPase (SERCA) sequesters Ca^2+^ in the endoplasmic reticulum to establish a reservoir for cell signaling. Because of its central importance in physiology, the activity of this transporter is tightly controlled *via* direct interactions with tissue-specific regulatory micropeptides that tune SERCA function to match changing physiological conditions. In the heart, the micropeptide phospholamban (PLB) inhibits SERCA, while dwarf open reading frame (DWORF) stimulates SERCA. These competing interactions determine cardiac performance by modulating the amplitude of Ca^2+^ signals that drive the contraction/relaxation cycle. We hypothesized that the functions of these peptides may relate to their reciprocal preferences for SERCA binding; SERCA binds PLB more avidly at low cytoplasmic [Ca^2+^] but binds DWORF better when [Ca^2+^] is high. In the present study, we demonstrated this opposing Ca^2+^ sensitivity is due to preferential binding of DWORF and PLB to different intermediate states that SERCA samples during the Ca^2+^ transport cycle. We show PLB binds best to the SERCA E1-ATP state, which prevails at low [Ca^2+^]. In contrast, DWORF binds most avidly to E1P and E2P states that are more populated when Ca^2+^ is elevated. Moreover, FRET microscopy revealed dynamic shifts in SERCA–micropeptide binding equilibria during cellular Ca^2+^ elevations. A computational model showed that DWORF exaggerates changes in PLB–SERCA binding during the cardiac cycle. These results suggest a mechanistic basis for inhibitory *versus* stimulatory micropeptide function, as well as a new role for DWORF as a modulator of dynamic oscillations of PLB–SERCA regulatory interactions.

The type 2a sarco/endoplasmic reticulum (ER) Ca^2+^-ATPase (SERCA2a) is a P-type ion transporter responsible for sequestering cytoplasmic Ca^2+^ into the sarcoplasmic reticulum (SR) of cardiac muscle cells. The rate of Ca^2+^ transport by SERCA2a determines how quickly the heart muscle relaxes during the diastolic phase of the cardiac cycle as the ventricle is filling with blood. SERCA2a function also sets the amplitude of SR Ca^2+^ release, which determines the heart’s contractile strength during the systolic phase when blood is being ejected from the heart. Pathological decreases in SERCA expression, function, and regulation are associated with heart failure (1, 2), focusing attention on SERCA as a possible therapeutic target (3, 4). So far, attempts to enhance transport function in patients by increasing expression of SERCA2a with gene delivery have been unsuccessful (5). Therefore, there is great interest in understanding physiological SERCA regulatory mechanisms to create a path toward rationally designed therapies that improve the function of the endogenous SERCA in patients with heart failure.

The principal regulator of SERCA function in the heart is phospholamban (PLB), a single-pass transmembrane micropeptide (Fig. 1, blue). PLB physically interacts with SERCA and reduces its apparent affinity for Ca^2+^, decreasing Ca^2+^ uptake (6). This inhibition is relieved at high concentrations of cytoplasmic Ca^2+^ and after phosphorylation of PLB by cAMP-dependent PKA (7, 8), providing a mechanism to increase Ca^2+^ transport in response to exercise and other physiologic stress. Initially, the relief of SERCA inhibition was thought to require dissociation of the PLB–SERCA complex after PLB phosphorylation or Ca^2+^ binding to SERCA (9, 10, 11, 12, 13). However, subsequent studies showed that the PLB–SERCA interaction can still occur after PLB phosphorylation or in high Ca^2+^ (14, 15), suggesting that PLB acts more like a subunit of a persistent SERCA regulatory complex. These seemingly alternative models may be reconciled by observations that SERCA binds PLB with modestly reduced affinity in elevated Ca^2+^ (16, 17, 18). PLB must bind more tightly to SERCA conformations that predominate at low Ca^2+^ and bind less avidly to SERCA states that prevail at elevated Ca^2+^. However, the energetics of PLB binding to specific SERCA conformers has not been measured. In the present study, we quantified PLB–SERCA interactions in intact cell membranes using FRET. Our goal was to compare the affinity of PLB for different intermediate states in the Ca^2+^ transport cycle.

**Figure 1:**
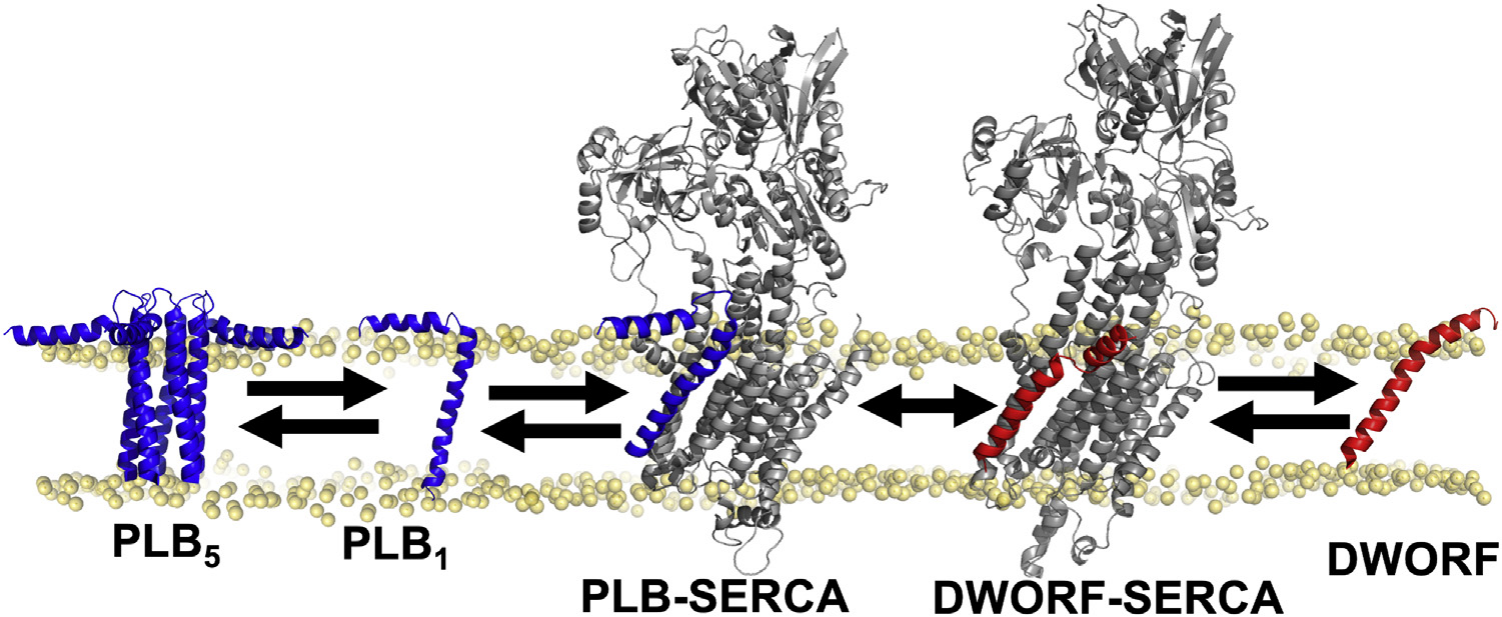
**Regulatory interactions of SERCA (*gray*) with PLB (*blue*) and DWORF (*red*).** Structures: PLB_5_, PDB:2KYV (59); PLB_1_, PDB:1FJP, (60); PLB-SERCA, DWORF–SERCA (18, 61); DWORF-7MPA, (25). DWORF, dwarf ORF; PDB, Protein Data Bank; PLB, phospholamban; SERCA, sarco/endoplasmic reticulum Ca^2+^-ATPase.

In addition to direct regulation by phosphorylation, PLB inhibitory potency may be modulated indirectly by oligomerization of PLB into pentamers (Fig. 1, “PLB_5_”). PLB pentamers represent an inactive, reserve pool (19) that serves as a buffer, reducing and stabilizing the concentration of active monomers. This buffering effect may be enhanced by adrenergic stimulation, since PLB pentamers are further stabilized by PLB phosphorylation (20, 21, 22). The rates at which PLB exchanges between SERCA- and pentamer-bound pools must govern how quickly these complexes can redistribute *in vivo*. We (23) and others (24) have provided evidence that the exchange of PLB monomers from pentamers occurs slowly relative to rapid exchange from the SERCA regulatory complex, but the underlying kinetics of these binding events have not been definitively measured. Therefore, the degree to which these regulatory complexes may dynamically redistribute in the cardiac SR remains unclear.

PLB competes for SERCA binding with another membrane micropeptide expressed in the heart, dwarf open reading frame (DWORF) (25), shown in *red* in Figure 1. In contrast to PLB, DWORF directly stimulates SERCA activity (18, 26) and displaces the inhibitory PLB (27, 28, 29, 30). Interestingly, our recent work showed that DWORF–SERCA affinity shows an opposite Ca^2+^-dependence compared to PLB–SERCA. That is, DWORF affinity for SERCA increases with elevated Ca^2+^ (18). This implies dynamic competition of stimulatory and inhibitory micropeptides: Ca^2+^ elevations favor DWORF binding to SERCA, but PLB becomes more competitive as Ca^2+^ levels fall. Here, we measured the dynamic binding of DWORF and PLB to SERCA during cellular Ca^2+^ oscillations to understand how the exchange of micropeptides may be important for dynamically responsive regulation. We have previously used FRET to examine shifts in the PLB–SERCA binding equilibrium in paced cardiac myocytes (16). However, motion artifacts and competing, non-FRET interactions with endogenous PLB/SERCA prevented a detailed analysis of exchange and equilibration rates in that study. To circumvent those experimental barriers we exploited a more well-controlled model system that mimics cardiac calcium handling. We interpreted these observations with a computational model that incorporates experimentally measured rate constants. This reductionist strategy yielded mechanistic insight into how regulatory equilibria shift during the cardiac cycle, and, on a longer timescale, how Ca^2+^ handling may adapt between rest and exercise. The results may inform future efforts to develop therapeutic strategies based on gene delivery of micropeptides (27, 30, 31).

## Results

### SERCA affinity for PLB and DWORF is dependent on Ca^2+^-pump conformation

**Figure 2:**
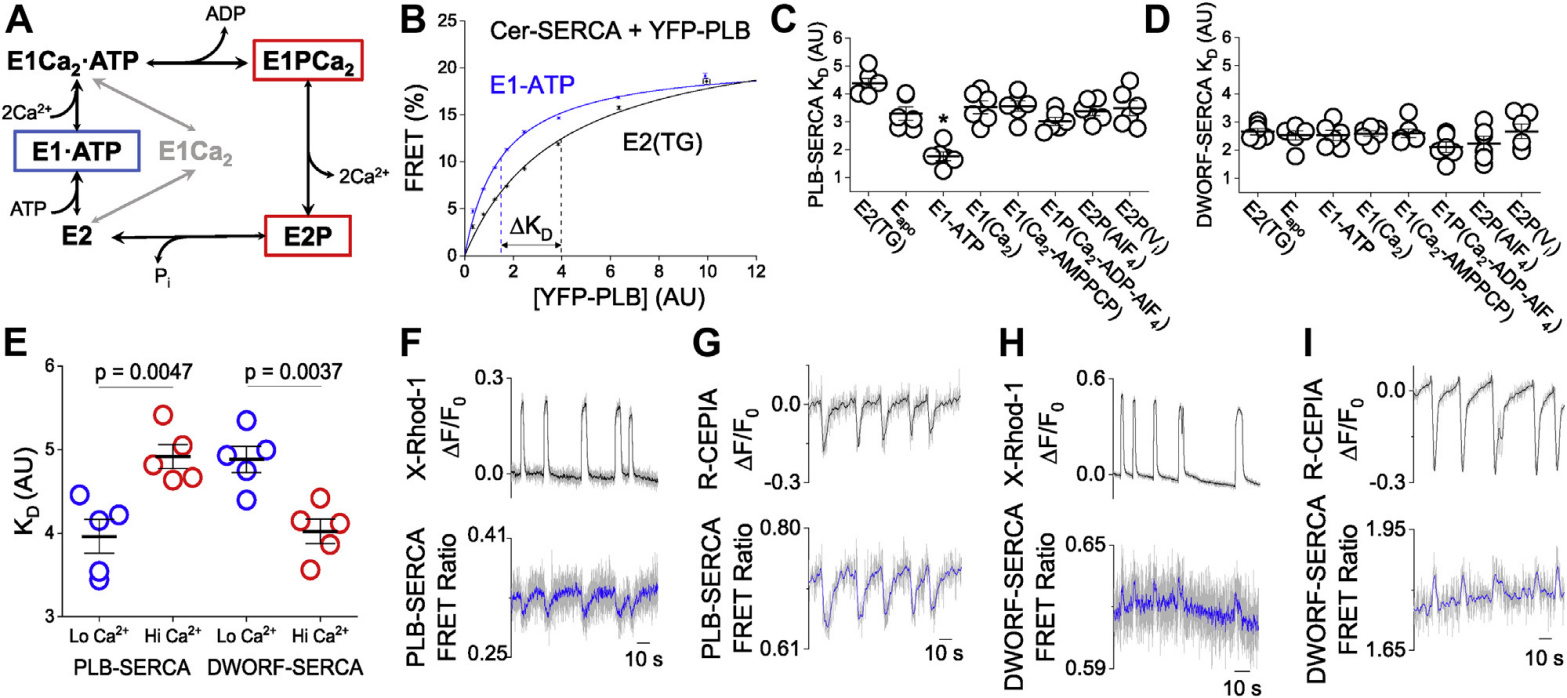
**Dynamics of PLB and DWORF binding to SERCA during elevations in intracellular Ca**^**2+**^**.***A*, a simplified Post-Albers scheme of the SERCA enzymatic cycle, highlighting states that predominate at low (*blue*) and high (*red*) intracellular [Ca^2+^]. *B*, FRET-based binding curves displaying a shifted dissociation constant (*K*_*D*_) of PLB–SERCA binding between the ATP-bound (*blue*) and TG-bound (*black*) states of SERCA. *C* and *D*, apparent *K*_*D*_s of PLB or DWORF binding to different SERCA enzymatic states of the catalytic cycle as in panel (*A*) with *lines* representing mean ± SEM (n = 6). Ligands used to stabilize each state are shown in *parentheses*. Differences in micropeptide *K*_*D*_s between SERCA states were analyzed by one-way ANOVA with Tukey’s post hoc (∗*p* < 0.05). *E*, apparent *K*_*D*_s of PLB and DWORF for SERCA in ATP-containing solutions with low (*blue*) and high (*red*) concentrations of intracellular Ca^2+^ with *lines* representing mean ± SEM (n = 5). Differences in *K*_*D*_ evaluated by Student’s *t* test. *F*, confocal microscopy quantification of intracellular Ca^2+^ measured by X-rhod-1 fluorescence (*gray* raw data, with *black smoothed trendline*) with simultaneous measurement of changes in PLB–SERCA FRET (YFP/Cer ratio) (*gray* raw data, with *blue smoothed trendline*). *G*, quantification of ER luminal Ca^2+^ measured by R-CEPIA1er fluorescence with simultaneous measurement of PLB–SERCA FRET (YFP/Cer ratio). *H*, quantification of intracellular Ca^2+^ measured by X-rhod-1 fluorescence with simultaneous measurement of DWORF–SERCA FRET (YFP/Cer ratio) *I*, quantification of ER luminal Ca^2+^ measured by R-CEPIA1er fluorescence with simultaneous measurement of DWORF–SERCA FRET (YFP/Cer ratio). DWORF, dwarf ORF; ER, endoplasmic reticulum; PLB, phospholamban; SERCA, sarco/endoplasmic reticulum Ca^2+^-ATPase.

We previously demonstrated that PLB–SERCA binding affinity is reduced in response to a sustained elevation of intracellular Ca^2+^, whereas the DWORF–SERCA interaction is slightly more stable with elevated [Ca^2+^] (16, 18). This reciprocal Ca^2+^ dependence prompted the hypothesis that PLB and DWORF may preferentially bind to different intermediate conformations of the SERCA enzymatic cycle. The SERCA enzymatic cycle (32) is represented in Figure 2*A*. At basal Ca^2+^ (low nM), SERCA predominantly resides in the ATP-bound state, E1-ATP (33) (Fig. 2*A*, *blue box*), waiting for Ca^2+^ to bind. During intracellular Ca^2+^ elevations, SERCA samples all the intermediate states of the Ca^2+^ transport cycle, but these states are not all equally populated during systole. Rather, there is relative accumulation in the autophosphorylated E1P/E2P intermediates that precede rate-limiting steps in its enzymatic cycle (34, 35). Those states are outlined with *red boxes* in Figure 2*A*.

To determine the relative affinity of both micropeptides for these and other key SERCA enzymatic states, we transfected HEK-293 cells with Cerulean (Cer)-labeled SERCA2a (FRET donor) and YFP-labeled PLB (FRET acceptor) and quantified the interaction of these proteins using acceptor sensitization FRET microscopy. Protein expression levels in membrane fractions prepared from HEK cells were comparable to protein concentrations in membranes prepared from human heart tissues (Fig. S1). To control the conformational poise of SERCA, cells were permeabilized with 0.05 mg/ml saponin in bath solutions appropriate for stabilization of the transporter in various conformations (see Experimental procedures and Fig. S2). The affinity of the PLB–SERCA interaction was quantified by measuring FRET with automated fluorescence microscopy, as previously described (20, 28). FRET was low in cells with low fluorescence (low protein expression), increasing to a maximum in the brightest cells (high protein expression), yielding a FRET based “binding curve” (Fig. 2*B*). A hyperbolic fit to the data yielded the maximal FRET efficiency at high protein concentration or FRET_max_ (representing the intrinsic FRET of the bound PLB–SERCA complex) and the apparent dissociation constant (*K*_*D*_), the protein concentration that yields half-maximal FRET efficiency. The *K*_*D*_ value is inversely related to the affinity of the PLB–SERCA complex. Figure 2*B* shows the conditions that yielded the greatest difference in relative *K*_*D*_ for the PLB regulatory complex. PLB bound to the E1-ATP state of SERCA is obtained with a solution containing 3 mM ATP and low Ca^2+^ and represents the prevailing conformation in resting (diastolic) conditions. Addition of thapsigargin (TG) resulted in a significant right-shift of the binding curve (*p* = 8 × 10^−3^) (Table S1), indicating a decrease in PLB–SERCA affinity. Reduced affinity of PLB for TG-inhibited SERCA is consistent with previous observations from our lab (16) and others (13).

The summary of fitting of binding curves obtained from six independent transfections for each of eight conditions is shown in Figure 2, *C* and *D* and Table 1 (Figs. S3 and S4). We observed especially avid binding of PLB to the E1-ATP state, which may indicate stabilization of that conformation of SERCA. This is the state that most SERCA pumps are in during the diastolic phase of the cardiac cycle when cytoplasmic Ca^2+^ is low, the muscle is relaxed, and the heart is filling with blood. The affinity of PLB for this state was not altered over a range of pH from 6 to 8 (Fig. S5), indicating no preference for “E2” or “E1” transmembrane domain conformations stabilized in acidic or alkaline pH, respectively (36, 37).

**Table 1:** Acceptor-sensitization FRET-binding curve parameters

Enzymatic state	Stabilizing ligand(s)	PLB-SERCA		DWORF–SERCA	
		*K*_*D*_ (AU)	FRET_max_ (%)	*K*_*D*_ (AU)	FRET_max_ (%)
E2	TG	4.4 ± 0.2	26 ± 0.8	2.7 ± 0.1	28 ± 0.5
E_apo_		3.3 ± 0.2	25 ± 0.9	2.5 ± 0.2	29 ± 1.1
E1-ATP	ATP	1.8 ± 0.2∗	22 ± 0.3	2.5 ± 0.2	29 ± 1.4
E1	Ca^2+^	3.5 ± 0.2	26 ± 0.9	2.6 ± 0.1	29 ± 0.7
E1	Ca^2+^-AMPPCP	3.6 ± 0.2	26 ± 0.8	2.6 ± 0.2	29 ± 1.6
E1P	Ca^2+^-ADP-AlF_4_	3.0 ± 0.1	22 ± 0.9	2.1 ± 0.2	25 ± 0.8
E2P	AlF_4_	3.4 ± 0.2	24 ± 0.9	2.2 ± 0.3	27 ± 1.0
E2P	V_i_	3.5 ± 0.3	26 ± 0.8	2.7 ± 0.3	31 ± 2.1

Data are reported as mean ± SEM (n = 6). Differences were determined by one-way ANOVA with Dunn’s post hoc (∗*p* < 0.05, See Table S1 for complete statistical analysis).

In contrast to PLB, DWORF showed a much flatter affinity profile across SERCA enzymatic states (Fig. 2*D*). We noted slightly lower *K*_*D*_ values for E1PCa and E2P states. The population of SERCA pumps increasingly accumulates in these states during the systolic phase of the cardiac cycle when cytoplasmic Ca^2+^ is elevated and the heart is contracting to eject blood. DWORF may have a preference for those conformations, though the difference was not statistically significant by one-way ANOVA (Tables 1 and S1). We did resolve statistically significant differences in PLB–SERCA and DWORF–SERCA affinities in a simpler comparison of affinities in ATP-containing solutions: low Ca^2+^ “relaxing solution” *versus* a high Ca^2+^ solution that induced enzymatic cycling of SERCA (see Experimental procedures). In these physiological buffers mimicking diastole *versus* systole, respectively, we observed reciprocal Ca^2+^-dependent binding affinity for PLB–SERCA *versus* DWORF–SERCA (Figs. 2*E* and S6), consistent with our previous study (18).

Taken together, these data indicate that PLB and DWORF prefer to bind different SERCA conformations corresponding to different enzymatic intermediate states of the transport cycle. To determine how this differential Ca^2+^-dependent affinity may cause dynamic shifts in PLB and DWORF binding equilibria during intracellular Ca^2+^ elevations, we exploited a cardiomimetic model system described previously (38). HEK-293 cells expressing RyR and SERCA2a show spontaneous ER Ca^2+^ release events that give rise to large, prolonged increases in cytoplasmic Ca^2+^. Ca^2+^ transients were detected by confocal microscopy as an increase in X-rhod-1 cytoplasmic Ca^2+^ indicator fluorescence or a decrease in R-CEPIA1er ER Ca^2+^ indicator fluorescence (Fig. 2, *F*–*I*, *black*). In some cells, these Ca^2+^ elevations coincided with small decreases in PLB–SERCA FRET, shown in Figure 2, *F* and *G* as *gray* data overlaid with a smoothed trendline (*blue*, see Experimental procedures) corresponding to a loss of PLB–SERCA affinity at elevated Ca^2+^. These were very small changes in FRET, as expected from the small differences in affinity quantified in equilibrium experiments (Fig. 2, *C* and *E*). Indeed, we were previously unable to detect a PLB–SERCA FRET change over a single Ca^2+^ transient in electrically paced cardiac myocytes, only observing a modest decrease in average FRET over a period of repeated Ca^2+^ transients during rapid pacing (16). However, in HEK cells the Ca^2+^ elevations were more prolonged (increasing time for quantification), expression of endogenous SERCA/PLB was very low (reducing competing non-FRET interactions), and the cells are noncontractile (eliminating confounding cell motions). These advantages enabled detection of small shifts in the binding equilibria in the present study.

Next, we tested whether there was a change in DWORF–SERCA FRET in response to Ca^2+^ elevations. These FRET changes were even smaller than those observed for PLB–SERCA binding (Fig. 2*H*), consistent with DWORF’s relatively flat affinity profile across SERCA enzymatic states (Fig. 2*D*). The DWORF–SERCA FRET change was easier to appreciate in experiments where a low affinity Ca^2+^ indicator localized to the ER lumen (R-CEPIA1er) was used instead of a cytoplasmic Ca^2+^ indicator dye (Fig. 2*I*), perhaps because ER Ca^2+^ buffering by the indicator resulted in larger amplitude Ca^2+^ release events. Interestingly, the direction of the DWORF–SERCA FRET change was the opposite of that observed for PLB–SERCA; we observed modestly increased FRET during Ca^2+^ transients (Fig. 2, *H* and *I*), consistent with enhanced DWORF–SERCA binding affinity at elevated cytoplasmic Ca^2+^ (Fig. 2*E*) (18).

### PLB reassociation with SERCA after Ca^2+^ transients is delayed by the PLB pentamer

**Figure 3:**
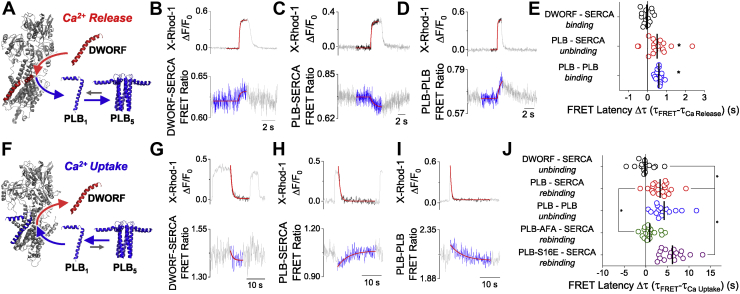
**PLB reassociation with SERCA after Ca**^**2+**^**transients are delayed by slow dissociation from the PLB pentamer.***A*, schematic diagram of shifts in PLB and DWORF binding equilibria during Ca^2+^ release. *B*, representative single exponential decay fit of the kinetics of DWORF–SERCA binding during Ca^2+^ release. *C*, kinetics of PLB–SERCA unbinding during Ca^2+^ release. *D*, kinetics of PLB–PLB binding during Ca^2+^ release. *E*, the latency of FRET ratio changes compared to Ca^2+^ release with *lines* representing mean ± SEM. Differences determined by one-way ANOVA with Dunn’s post hoc test (∗*p* < 0.05, see Table S4 for complete statistical analysis). *F*, schematic diagram of shifts PLB and DWORF binding equilibria during Ca^2+^ uptake. *G*, representative single exponential decay fit of the kinetics of DWORF–SERCA unbinding during Ca^2+^ uptake. *H*, kinetics of PLB–SERCA rebinding during Ca^2+^ uptake. *I*, kinetics of PLB–PLB binding during Ca^2+^ release. *J*, the latency of FRET ratio changes compared to Ca^2+^ release with *lines* representing mean ± SEM. Differences determined by one-way ANOVA with Dunn’s post hoc test (∗*p* < 0.05, see Table S7 for complete statistical analysis). DWORF, dwarf ORF; PLB, phospholamban; SERCA, sarco/endoplasmic reticulum Ca^2+^-ATPase.

To examine how the kinetics of cellular Ca^2+^ signaling determines SERCA–micropeptide binding dynamics, we quantified the rates of change of FRET and [Ca^2+^]. Specifically, exponential decay fitting of these changes revealed characteristic time constants (τ) for each process. Analysis of the Ca^2+^ release process (Fig. 3*A*) showed that DWORF–SERCA binding occurred with a time course that was similar to the rate of rise of Ca^2+^ (τ = 0.22 ± 0.03 s and 0.21 ± 0.03 s, respectively) (Fig. 3*B*). In contrast, PLB–SERCA unbinding was slower (τ = 0.74 ± 0.14 s), lagging behind the Ca^2+^ upstroke (*p* = 4 × 10^−8^) (Fig. 3*C*). In addition, we evaluated how Ca^2+^-dependent changes in PLB–SERCA binding affects the PLB monomer–pentamer equilibrium, measuring intrapentameric FRET between Cer-PLB and YFP-PLB. Interestingly, PLB–PLB FRET increased rapidly during Ca^2+^ elevations (Fig. S6). The inverse changes in PLB–SERCA FRET and PLB–PLB FRET show that as PLB is displaced from SERCA, it is rapidly incorporated into pentamers. The time course of PLB–PLB binding (τ = 0.59 ± 0.04 s) also lagged slightly behind Ca^2+^ release (*p* = 2 × 10^−4^) (Fig. 3*D*). This delay was similar to that of PLB–SERCA unbinding (Fig. 3*E*), suggesting that PLB–SERCA unbinding may be rate limiting for subsequent PLB oligomerization during Ca^2+^ elevations. The kinetics of SERCA–micropeptide binding dynamics during Ca^2+^ release are summarized in Fig. S8 and Tables S2–S4.

Next, we examined changes in SERCA–micropeptide binding as Ca^2+^ declined to baseline during the Ca^2+^-reuptake phase of the Ca^2+^ transient (Fig. 3*F*). DWORF dissociated rapidly from SERCA, as measured by the decrease in the DWORF–SERCA FRET ratio back to baseline (Fig. 3*G*). This unbinding process occurred with a τ of 1.2 ± 0.4 s, similar to the Ca^2+^ transient relaxation time. However, PLB–SERCA reassociation displayed a significant lag compared to the Ca^2+^ transient relaxation, with FRET continuing to increase back toward maximum for several seconds after Ca^2+^ had already stabilized at a basal level (Fig. 3*H*). PLB–SERCA reassociation occurred with a τ of 4.9 ± 0.6 s, much slower than the Ca^2+^ relaxation time (τ = 1.6 ± 0.2 s, *p* = 2 × 10^−8^).

To understand this unexpectedly slow rate for PLB–SERCA rebinding, we considered whether the rate of PLB–SERCA recovery could be limited by slow dissociation of PLB monomers from the PLB pentamer (23, 24). Indeed, PLB–PLB FRET relaxation (unbinding of monomers from the pentamer) displayed a slow time course (τ = 5.2 ± 0.8 s) (Fig. 3*I*) that closely matched the slow PLB–SERCA reassociation (4.9 ± 0.6 s) (Fig. 3*J*). The data suggest PLB pentamer dissociation is rate limiting for PLB rebinding SERCA after Ca^2+^ elevations. This interpretation was further supported by the observation that destabilization of the PLB pentamer by mutagenesis (PLB-AFA) (39) accelerated the PLB–SERCA reassociation rate (τ = 2.5 ± 0.5 s, *p* = 8 × 10^−3^), such that the FRET change no longer lagged behind the Ca^2+^ transient decay (Figs. 3*J* and S9).

We also examined how pentamer dissociation kinetics could be physiologically tuned to control the PLB–SERCA reassociation rate. Equilibrium measurements of PLB oligomerization have shown that PLB pentamers are stabilized by PKA phosphorylation of serine 16 of PLB (21), which occurs as an outcome of adrenergic signaling during physiological stress. Here, we found that stabilization of pentameric interactions by a phosphomimetic S16E mutation further slowed the rate of PLB–SERCA reassociation (τ = 8.6 ± 0.7 s, *p* = 2 × 10^−6^) (Figs. 3*J* and S10). The kinetics of SERCA–micropeptide binding dynamics during Ca^2+^ uptake are summarized in Fig. S11 and Table S5–S7. Overall, the results support the hypothesis that PLB pentamers delay reassociation of the dynamic fraction of PLB with SERCA after Ca^2+^ elevations, and this physiological mechanism is tuned under the control of adrenergic signaling.

### Modeling the redistribution of PLB and DWORF regulatory complexes

**Figure 4:**
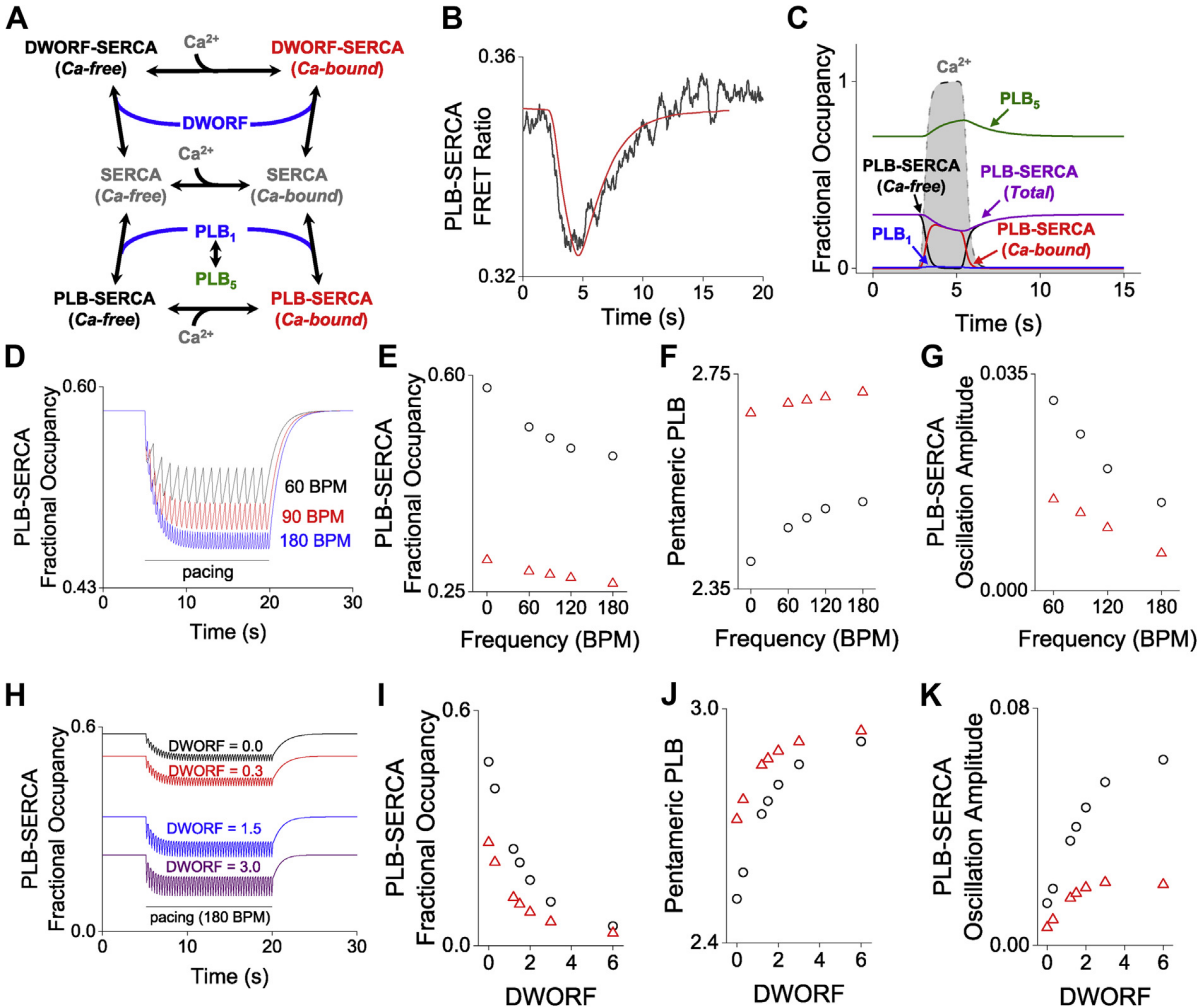
**A computational model simulated the dynamics of PLB and DWORF interactions with SERCA.***A*, simplified diagram of modeled regulatory interactions. *B*, a fit of the model to a representative FRET change measured by confocal microscopy. *C*, simulation of changes in the populations of regulatory species during a Ca^2+^ elevation, where relative amounts of SERCA and PLB are equal. *Black circles* represent the results of the model of the normal, nonfailing heart. *Red triangles* represent adjustment of the model to simulate heart failure. *D*, simulation of the effect of cardiac pacing on PLB–SERCA at three heart rates (beats per min, BPM), where the ratio of SERCA:PLB was 1:3. *E*, increasing pacing frequency modestly decreased PLB–SERCA binding. *F*, increasing pacing frequency increased PLB oligomerization. *G*, PLB–SERCA oscillation amplitude decreased with faster pacing. *H*, simulation of the effect of increasing DWORF expression relative to SERCA on PLB–SERCA binding. *I*, increasing DWORF resulted in a decrease in the equilibrium level of PLB–SERCA. *J*, increasing DWORF increased PLB oligomerization. *K*, increasing DWORF relative to SERCA resulted in larger oscillations in PLB–SERCA binding. For *H*–*K*, the ratio of SERCA:PLB was 1:3 and pacing rate was 180 BPM. DWORF, dwarf ORF; PLB, phospholamban; SERCA, sarco/endoplasmic reticulum Ca^2+^-ATPase.

To interpret our FRET-based measurements of SERCA–micropeptide binding dynamics, we developed a computational model to integrate the measured rates of the dynamic interactions of PLB and DWORF with SERCA in the context of the human cardiac cycle. Using this model, we simulated the dynamic redistribution of these regulatory complexes between systole and diastole, or on a longer timescale, between rest and exercise. The model describes the kinetics of these regulatory interactions with a set of numerically solved ordinary differential equations (see Supplemental Methods). A genetic algorithm was used to fit mean rate constants for the forward and reverse reactions in the model from experimentally measured FRET data as previously described (40). For simplicity, we considered the population of SERCA pumps to be distributed between two ensembles of pump enzymatic states, representing the “diastolic” condition (Ca-free) and the “systolic” condition (Ca-bound), as diagrammed in Figure 4*A*. The relative population of these two ensemble states is dependent on the relative concentration of Ca^2+^, as quantified by confocal microscopy experiments. Monomeric PLB (PLB_1_) can bind either the Ca-free or Ca-bound ensemble of SERCA (16) but with higher affinity for the Ca-free population (Fig. 2*E*). The relative affinity of PLB for SERCA was constrained to be twofold higher for Ca-free *versus* Ca-bound ensembles, as determined experimentally from FRET-binding curves (Fig. 2*C*) (18). Monomeric PLB (PLB_1_) is also in equilibrium with the pentameric population (PLB_5_). The relative affinity of DWORF for SERCA was constrained to increase by 25% between Ca-free and Ca-bound ensembles, consistent with FRET measurements (Fig. 2*D*) (18). The computed kinetic parameters are provided in the Supplemental data (Fig. S12).

### Dynamic responses to Ca^2+^ transients

The computational model provided good descriptions of data from physical experiments showing time-dependent changes in PLB–SERCA FRET and cytoplasmic Ca^2+^ (measured simultaneously). Figure 4*B* provides a best-fit model prediction of the FRET compared to a representative experimental measurement. The model captured the rapid reduction of FRET with the rise of the Ca^2+^ transient. The decrease in PLB–SERCA is followed by a slower restoration of the FRET as Ca^2+^ returns to baseline. The similarity of the predicted and experimentally measured FRET data suggests that the model appropriately represents key aspects of PLB–SERCA binding dynamics.

Using these fit parameter values, we simulated the time-dependent changes in PLB–SERCA and PLB–PLB binding dynamics that we observed in HEK cells. Importantly, we observed that a majority of the PLB–SERCA regulatory complexes remained intact (Fig. 4*C*, *purple*) during a Ca^2+^ elevation (Fig. 4*C*, *gray shaded region*), consistent with the “subunit model” (41). Thus, PLB can remain bound to SERCA when SERCA is bound to Ca^2+^; this Ca^2+^-bound PLB–SERCA fraction is highlighted in Figure 4*C* (*red*). However, a fraction of the population of PLB–SERCA dissociated during a Ca^2+^ transient (Fig. 4*C*, *purple*). This fraction comprised ∼30% of PLB–SERCA complexes under these simulation conditions. This shift impacted the PLB monomer/pentamer equilibrium (Fig. 4*C*, *blue*/*green*). Expectedly, there was very little observed change in the small population of PLB monomers, as the dynamic fraction was quickly incorporated into pentamers. These reciprocal shifts are the cause of the transient decrease in SERCA–PLB FRET and increase in PLB–PLB FRET observed by confocal microscopy (Fig. 3, *C* and *D*).

Next, we simulated the shorter, faster Ca^2+^ transients observed in the heart, pacing at a range of frequencies from 60 to 180 beats per minute, to investigate regulatory dynamics under conditions of rest, moderate exercise, and intense exercise. Increasing pacing frequency progressively decreased PLB–SERCA binding (Fig. 4, *D* and *E*, *black circles*) and increased accumulation of PLB in pentamers (Fig. 4*F*, *black circles*). The systole–diastole difference in PLB–SERCA binding was small. This oscillation became even smaller with increasing pacing frequency (Fig. 4, *D* and *G*, *black circles*), as the shifts in binding equilibria began to lag behind the rapid Ca^2+^ changes. These results suggest that the exchange dynamics of PLB may impact adaptive SERCA regulation between resting and exercising heart rates.

To assess how PLB–SERCA binding dynamics may be altered in disease, we modified the model conditions to reflect changes observed in heart failure. Specifically, the SERCA population was reduced by 40% (2) and diastolic Ca^2+^ was increased by 50% (40). The heart failure condition is shown in Figure 4 as *red triangles*. As expected, the heart failure condition decreased the population of PLB bound to SERCA at equilibrium (Fig. 4*E*), decreased PLB–SERCA oscillation amplitude (Fig. 4*G*), and blunted the responsiveness of these parameters to increasing heart rates (Fig. 4, *E*–*G*). This suggests frequency-dependent changes in PLB inhibition may be reduced in heart failure.

Introduction of DWORF–SERCA interactions to the model yielded the expected effect of competition of DWORF and PLB for SERCA binding (Fig. 4*A*). Increasing the amount of DWORF relative to PLB decreased PLB–SERCA binding (Fig. 4, *H* and *I*, *black circles*) and increased PLB oligomerization (Fig. 4*J*, *black circles*). DWORF competes potently with PLB even at low stoichiometry because oligomerization of PLB reduces the effective concentration of the active monomeric species. Interestingly, the Ca^2+^-dependent increase in DWORF affinity for SERCA exaggerated the oscillations in PLB–SERCA binding during pacing (Fig. 4*K*, *black circles*). That is, during Ca^2+^ elevations DWORF (which binds SERCA better at high Ca^2+^) increasingly displaced PLB (which binds SERCA better at low Ca^2+^).

Under conditions representing heart failure (decreased SERCA expression, increased diastolic Ca^2+^) (Fig. 4, *I*–*K*, *red triangles*), there was decreased formation of the PLB–SERCA complex (Fig. 4*E*), and therefore, the effect of DWORF competition was blunted (Fig. 4*I*). Moreover, since changes in PLB–SERCA binding between systole and diastole were smaller in heart failure, the ability of DWORF to exaggerate this change through calcium-dependent competition was also decreased (Fig. 4*I*). These results suggest that the impact of DWORF on PLB–SERCA binding dynamics may be reduced in heart failure.

## Discussion

### Differential Ca^2+^-dependence of micropeptides for SERCA

The principal finding of the present study is that PLB and DWORF preferentially bind to different conformations of SERCA (Fig. 2, *C* and *D*), and this is the underlying cause of the reciprocal Ca^2+^-dependent affinities of these micropeptides (Fig. 2*E*) (18). The FRET-based binding assay showed that PLB has the highest affinity for the E1-ATP state (Fig. 2*C*), which is the predominant state at resting Ca^2+^ (Fig. 2*A*, *blue box*). In contrast, DWORF shows a much flatter SERCA-binding profile, with more avid binding to E1P and E2P states (Fig. 2*D*). Much of the SERCA population accumulates in these states when cytoplasmic Ca^2+^ is elevated. This accumulation occurs because of rate-limiting, slow steps in the SERCA enzymatic cycle (34, 35), which cause rapidly cycling pumps to build up in “traffic jams” in E1P and E2P (Fig. 2*A*, *red boxes*). Figure 3, *A* and *F* show how differential binding of micropeptides leads to exchange of a dynamic fraction of PLB and DWORF from the binding site on SERCA during intracellular Ca^2+^ elevations. In high Ca^2+^ (Fig. 3*A*), DWORF increasingly outcompetes PLB, displacing a fraction of monomeric PLB (“PLB_1_”), which is increasingly incorporated into PLB pentamers (“PLB_5_”). Conversely, in low Ca^2+^ (Fig. 3*F*), DWORF binding is decreased, and PLB monomers increasingly bind to SERCA because the pump accumulates in the E1-ATP state that PLB prefers. This recovery process is rate limited by the slow dissociation of monomeric PLB from pentamers (Fig. 3*I*).

We propose that differential Ca^2+^ dependence is the key determinant of whether a micropeptide is inhibitory or stimulatory for SERCA function. In particular, micropeptide–SERCA binding affinity may be taken as an index of the relative energetics of the regulatory complex, much as melting temperature may be quantified as a proxy for the stability of a protein–protein complex (42). Thus, avid PLB binding to the E1-ATP structure (Fig. 2*A*, *blue box*) is expected to stabilize that complex and favor the population of that state of SERCA. The functional consequence of stabilizing this state is to slow the subsequent Ca^2+^-binding step in the enzymatic cycle. This may account for the principal inhibitory effects of PLB, reducing the apparent Ca^2+^ affinity of the pump and slowing pump turnover (6). Moreover, we propose that while PLB binding to E1-ATP deepens a depression in the energy landscape, DWORF reduces the height of a peak, lowering an energy barrier by stabilizing E1P/E2P states. Increasing pump flux through these rate-limiting steps enhances enzyme turnover when Ca^2+^ is high (18).

In addition, the dynamics of PLB and DWORF binding and unbinding from SERCA described here have important functional implications. Reciprocal binding may be important for reacting to transient Ca^2+^ changes during the cardiac cycle or, on a longer timescale, responding to adrenergic signaling and changes in pacing frequency during exercise. The present data provide insight into Ca^2+^ transport regulation on both timescales, as discussed in the following sections.

### Functional implications of micropeptide exchange during the cardiac cycle

The change in PLB–SERCA binding with Ca^2+^ is only approximately twofold, so PLB–SERCA complexes do not completely dissociate during systole (Fig. 4*D*) (16, 18). However, cardiac Ca^2+^ elevations simultaneously reduce PLB-binding affinity and favor DWORF binding, so a fraction of the population of these micropeptides must exchange from SERCA during each Ca^2+^ transient. Our computational model showed that the expected functional impact of this exchange is to exaggerate the intrinsic response of SERCA to changing Ca^2+^. That is, during diastole (cardiac relaxation), SERCA activity is already low because Ca^2+^ is low. SERCA is further inhibited in diastole by an increase in the fraction of pumps that bind PLB (Fig. 2, *F* and *G*). Then, during systole (cardiac contraction), Ca^2+^ is high, supporting high SERCA activity. This high SERCA activity is further enhanced by increased DWORF binding (Fig. 2, *H* and *I*), which increasingly displaces PLB and directly stimulates SERCA maximal activity (18, 25, 26, 27, 29, 30, 31). Together, low Ca^2+^ inhibition and high Ca^2+^ stimulation should enhance the apparent cooperativity of the Ca^2+^ response, which would benefit cardiac function by conserving ATP consumption until Ca^2+^ transport is most efficient and most needed. Thus, competitive binding of SERCA by DWORF decreases both the basal level of PLB–SERCA binding as previously proposed (29) and also increases the amplitude of the oscillations in the population of the PLB–SERCA complex (Fig. 4, *I*–*K*). The dose dependence of these effects revealed in the computational model may provide guidance for future DWORF-based gene therapy approaches (27, 30, 31).

### The function of the PLB pentamer kinetic trap in rest and exercise

The observations presented here also have implications for the enhancement of cardiac function during exercise. PLB dissociation from pentamers is slow (τ ∼5 s) (Fig. 3*D*), a noteworthy finding since this is significantly slower than the cardiac cycle. Even at a resting heart rate, a 1 s interval between beats would not be sufficient time for the equilibrium to fully relax between systole and diastole. Therefore, Ca^2+^ signals must also integrate over a longer timescale, such as between rest and exercise. This is visualized in our computational model, which showed increasing accumulation of PLB in pentamers at faster heart rates at the expense of the population of PLB–SERCA (Fig. 4*D*). Such “kinetic trapping” of PLB in pentamers at faster heart rates is further increased by phosphorylation of PLB by PKA or CaMKII, which stabilizes the pentamer complex (20, 21, 22). These kinases are activated in exercise through adrenergic signaling and prolonged Ca^2+^ elevation, respectively. The functional consequence of decreased SERCA inhibition by PLB is increased SR Ca^2+^ load and enhanced cardiac contractility. Thus, one may speculate that frequency-dependent accumulation of PLB in pentamers contributes to the Bowditch effect, a positive force–frequency relationship in which a faster heart rate causes more forceful contractions of the heart. The mechanism we propose may explain previous studies that showed the Bowditch effect was absent in PLB KO mice (43, 44, 45, 46). Moreover, simulations showed that under pathological conditions SERCA regulation is decreased and the frequency dependence of that regulation is diminished (Fig. 4, *E*–*G*). This may explain why the Bowditch effect is blunted in patients with heart failure (47).

### Summary and future directions

Dynamic changes in the competition of regulatory micropeptides on seconds and minutes timescales represent an important aspect of the responsiveness of SERCA regulation to Ca^2+^ transients. The present data clarify the role of the PLB pentamer as a phosphorylation-tunable kinetic trap that limits the rate of SERCA rebinding by a dynamic fraction of PLB. Future studies will test the functional predictions derived from the present results: DWORF enhances transitions through E1P, E2P states in the SERCA enzymatic cycle; DWORF increases the size of the fraction of PLB that binds/unbinds SERCA with each Ca^2+^ elevation, exaggerating the SERCA inhibition/stimulation cycle; DWORF and PLB act in concert to increase the cooperativity of Ca^2+^ transport. Overall, the data suggest that dynamic competition of PLB and DWORF is an important determinant of cardiac function. More generally, the results may provide insight into the interplay of other tissue-specific micropeptide regulators of ion transporters.

## Experimental procedures

### Plasmid constructs

For all plasmid constructs, we used pEGFP-C1 as the expression vector in mammalian cells. All micropeptides (PLB, DWORF, PLB-AFA, and PLB-S16A) and SERCA constructs consisted of mCerulean3, enhanced YFP, or TagRFP fused *via* a five amino acid linker to the N terminus of the micropeptide or SERCA (23, 27, 48). We previously showed SERCA singly or doubly labeled with fluorescent protein still retains normal ATPase activity (49, 50) and Ca^2+^ transport function (49, 51, 52). In addition, SERCA fused to two fluorescent proteins can be regulated by PLB fused to a third fluorescent protein (49). The data suggest that the tags are benign for the pump and regulator.

### Cell culture and transfection

AAV 293 cells were cultured in Dulbecco's modified Eagle's medium (DMEM) cell culture medium supplemented with 10% fetal bovine serum (ThermoScientific). Following culture, the cells were transiently transfected using either MBS mammalian transfection kit (Agilent Technologies, Stratagene) or Lipofectamine 3000 transfection kit (Invitrogen) as per instructions provided with the respective kits. Twenty-four hours post-transfection, the cells were trypsinized (ThermoScientific) and replated onto poly-D-lysine–coated glass bottom chambers and allowed to settle down for 1 h before imaging. We have previously estimated that this approach achieves ER protein expression levels on the order of 2000:1 or 4000:1 lipids:protein ratio (53), *versus* 750:1 lipid:protein ratio quantified for native SR (41, 54). Thus, native protein concentrations are threefold to fivefold more concentrated, and we do not consider this heterologous model to represent “overexpression” of SR proteins.

### Human heart tissue procurement

Human left ventricular tissue was provided by Loyola Cardiovascular Research Institute Biorepository. The sample collection was approved by Loyola University Review Board (IRB number 210940821918) and written informed consent was obtained for collection of heart tissue according to Declaration of Helsinki.

### Human tissue membrane protein enrichment

Membrane protein enriched fractions were prepared from human heart tissue as previously described (55). Briefly, frozen heart tissue was placed in 5 ml of a solution containing 100 mM KCl, 2.5 mM K_2_HPO_4_, 2.5 mM KH_2_PO_4_, 2 mM EDTA, and protease inhibitors. Samples were mechanically homogenized with an Omni International GLH-01 homogenizer and rotated for 1 h at 4 °C. Homogenates were centrifuged at 10,000*g* for 20 min at 4 °C. Supernatants were collected and centrifuged at 48,000*g* for 1 h at 4 °C. The pellet containing membrane fractions was resuspended in 100 μl of a solution containing 1 M sucrose and 50 mM KCl.

### HEK cell membrane protein enrichment

Membrane protein enriched fractions were prepared from transfected HEK cells as previously described (55). Fourty-eight hours post-transfection, HEK cells were scraped in 5 ml of homogenizing solution containing 250 mM sucrose, 10 mM Tris, and 2 mM EDTA pH 7.4 with protease inhibitors and centrifuged at 4000*g* for 10 min at 4 °C. Cell pellets were resuspended in homogenizing solution and mechanically homogenized. Cell homogenates were centrifuged at 4000*g* for 20 min at 4 °C. Supernatants were collected and centrifuged at 55,000*g* for 30 min at 4 °C. The pellet containing membrane fractions was resuspended in 100 μl of a solution containing 1 M sucrose and 50 mM KCl.

### Western blotting

Sample total protein concentrations were determined using a Pierce bicinchoninic acid protein assay kit. Thirty micrograms of sample were diluted in 4× Laemmli sample buffer with β-mercaptoethanol at 1:1 ratio, denatured at 90 °C for 5 min, run on a 4 to 15% polyacrylamide gradient gel, and transferred to a polyvinylidene difluoride membrane. The membrane was stained with Revert total protein stain (LI-COR Biosciences) for 5 min to obtain total protein in each lane and then blocked for 1 h at room temperature in Intercept blocking buffer (LI-COR Biosciences) diluted at a 1:1 ratio in PBS with Tween 20 (PBS-T). Blots were incubated overnight at 4 °C with primary antibody diluted 1:1000 in PBS-T: mouse anti-SERCA2 (abcam; ref# ab2817), mouse anti-PLB (Invitrogen; ref# MA3-922;2D12), or rabbit anti-DWORF (29) (a gift of Catherine Makarewich, Cincinnati Children’s Hospital). The anti-DWORF antibody was a custom polyclonal antibody derived against the N-terminal region of the mouse DWORF protein sequence (MAEKESTSPHLI) and did not react with human DWORF (Fig. S1). The blots were then incubated with antimouse (IRDye 680RD; LI-COR Biosciences) or anti-rabbit (IRDye 800CW; LI-COR Biosciences) secondary antibody diluted 1:10,000 in PBS-T. Blots were imaged using an Azure c600 gel imaging system and analyzed using the LI-COR Image Studio software.

### FRET acceptor sensitization in permeabilized HEK-293 cells

Acceptor sensitization FRET was quantified as previously described (16). Briefly, AAV 293 cells were transiently transfected with Cer-donor and YFP acceptor–labeled FRET-binding partners in a 1:5 M plasmid ratio. FRET acceptor sensitization was measured by automated fluorescence microscopy before and after permeabilization with 0.005% w/v saponin. For each condition, two sets of 72 images (∼500 total cells per condition) were collected from six independent experiments with a 20 × 0.75 numerical aperture objective with 50 ms exposure for each channel: Cer, YFP, and FRET (Cer excitation/YFP emission). Cells that expressed Cer had an area of 136 to 679 μm^2^ and were at least 40% circular, were automatically scored for Cer, YFP, and FRET fluorescence intensity with a rolling background subtraction using a plugin in Fiji (imagej.net/software/fiji/). FRET efficiency was calculated according to $E=G/\left(G+2.782\;\text{X }F_{Cer}\right)$, where $G=F_{FRET}-a\;\text{X }F_{YFP}-d\;\text{X }F_{Cer}$, where $F_{FRET}$, $F_{YFP}$, and $F_{Cer}$ are the fluorescence intensities from FRET, YFP, and Cer images, respectively, and *G* represents FRET intensity corrected for the bleedthrough of the channels. The parameters *a* and *d* are bleedthrough constants calculated as $a=F_{FRET}/F_{YFP}$ for a control sample transfected only with YFP-SERCA and $d=F_{FRET}/F_{Cer}$ for a control sample transfected only with Cer-SERCA. For our experimental setup, *a* and *d* were 0.185 and 0.405, respectively. FRET efficiency for each scored cell was plotted as a function of expressed protein concentration, as determined from the fluorescence intensity of the YFP channel (20, 28). FRET was low in cells with low fluorescence (low protein expression), increasing to a maximum in the brightest cells (high protein expression), yielding a FRET-based “binding curve.” The data were fit to a hyperbolic function of the form [FRET = FRET_max_ [YFP]/(K_D_+[YFP]), where FRET_max_ is the maximal FRET efficiency at high protein concentration (representing the intrinsic FRET of the bound complex), [YFP] is inferred from YFP fluorescence emission in each cell, and *K*_*D*_ is the apparent dissociation constant (the protein concentration that yields half-maximal FRET efficiency).

To control the conformational poise of SERCA, cells were permeabilized with 0.05 mg/ml saponin in bath solutions, appropriate for stabilization of the transporter in various conformations. Solutions for ligand stabilization of enzymatic intermediate states were prepared by addition of corresponding substrates to a calcium-free base solution, which includes 100 mM KCl, 5 mM MgCl_2_, 2 mM EGTA, and 10 mM imidazole, pH 7.0. The base solution was used to characterize SERCA in a ligand-free state, E_apo_. The following ligands were used to prepare specific solutions corresponding to SERCA biochemical state: 100 μM TG for E2(TG); 3 mM ATP for E1-ATP; 2.1 mM CaCl_2_ for E1(Ca_2_) with free [Ca^2+^]_i_ = 100 μM (56); 2.1 mM CaCl_2_ and 500 μM nonhydrolyzable ATP analog (AMPPCP) for E1(Ca_2_-AMPPCP); 2.1 mM CaCl_2_, 500 μM ADP, 50 μM AlCl_3_, and 3 mM KF for E1(Ca_2_–ADP–AlF_4_^−^); 0.1 mM orthovanadate for E2(Vi); and 50 μM AlCl_3_ and 3 mM KF for E2(AlF_4_^−^). Concentrations of AMPPCP nucleotide analog and ADP nucleotide were reduced from previously published conditions (3 mM) used to stabilize SERCA in microsomal fractions (27) in order to prevent altering fluorescent protein emission intensity. The data for each set of binding partners were analyzed for differences in *K*_*D*_ between buffer conditions using a one-way ANOVA with Tukey’s post hoc test (significance = *p* < 0.05).

For experiments measuring PLB-binding preference for SERCA enzymatic states E2-ATP and E1-ATP, which are in equilibrium under low Ca^2+^, high ATP conditions (*e.g.*, cardiac diastole), a buffer containing 100 mM KCl, 5 mM MgCl_2_, 2 mM EGTA, 10 mM imidazole, and 3 mM ATP was prepared. The pH was then adjusted to the following concentrations: pH = 6.0, 6.5, 7.0, 7.5, and 8.0. For these experiments, a TagRFP-PLB acceptor construct (p*K*_a_ = 3.8) was used in place of the YFP-PLB acceptor because YFP fluorescence is sensitive to pH changes in this range (p*K*_a_ = 6.9) (57).

For experiments with low and elevated intracellular [Ca^2+^], solutions were prepared containing potassium aspartate 120 mM, KCl 15 mM, KH_2_PO_4_ 5 mM, MgCl_2_ 0.75 mM, dextran 2%, ATP 5 mM, Hepes 20 mM, and EGTA 2 mM, pH 7.2. The elevated [Ca^2+^] buffer was prepared with CaCl_2_ 1.7 mM for a free [Ca^2+^]_i_ = 2 μM (56). The data for each set of binding partners were analyzed for differences in *K*_*D*_ between high and low Ca^2+^ conditions using a Student’s *t* test (significance = *p* < 0.05).

### Confocal fluorescence microscopy to measure intermolecular FRET and HEK-293 cytoplasmic Ca^2+^

HEK-293 cells exhibiting spontaneous Ca^2+^ oscillations were generated by transient transfection with GFP-tagged RyR2 and either Cer or unlabeled SERCA2a and cotransfected with SERCA, PLB, PLB-AFA, PLB-S16E, or DWORF FRET pairs tagged with Cer and YFP fluorophores. Transfected cells were cultured for 24 h and seeded into poly-D-lysine–coated glass bottom chamber slides in DMEM plus 10% fetal bovine serum. Twenty-four hours after seeding, cell culture medium was changed with PBS (+Ca^2+^/+Mg^2+^), and experiments were conducted with a Leica SP5 laser scanning confocal microscope equipped with a 63× water objective. To observe transient changes in cytoplasmic calcium, cells were incubated with 10 μM X-Rhod-1/AM (X-Rhod) for 20 min in PBS (+Ca^2+^/+Mg^2+^). X-Rhod was excited with the 543 nm line of a He-Ne laser, and emitted fluorescence was measured at wavelength 580 nm. FRET pair fluorophores Cer and YFP were excited with the 430 and 514 nm lines of an argon laser, respectively, and emitted fluorescence was measured at wavelengths 485 ± 15 and 537 ± 15 nm, respectively. Images were acquired in line scan with averaging of four every 134 ms for ∼2 min.

A select set of concurrent experiments were conducted with a Zeiss LSM 880 Airyscan confocal microscope using a 40× oil immersion objective. X-Rhod was excited with the 594 nm line of a He-Ne laser, and emitted fluorescence was measured at wavelength 580 nm. FRET pair fluorophores Cer and YFP were excited with the 458 nm line of an argon laser, and emitted fluorescence was measured at wavelengths 485 ± 15 and 537 ± 15 nm, respectively. Images were acquired in line scan every 24 ms for ∼2 min. This faster acquisition rate was used to resolve time-dependent changes in FRET signals during the fast upstroke of cellular Ca^2+^ elevations.

FRET ratio was determined by dividing the acceptor fluorescence by the donor fluorescence and plotted as a function of time with X-Rhod to indicate concurrent changes in [Ca^2+^]. FRET ratio data was smoothed using a Savitzky–Golay binomial filter with a 4.08 s averaging window. Changes in FRET ratio and X-Rhod fluorescence associated with Ca^2+^ uptake were fit to the single-exponential decay function, $y=A1^{-x/\tau }+y_{0}$ in origin, to estimate the time constant or τ of the change, where A1 is the amplitude of change and $y_{0}$ is the initial detected fluorescence. Changes in FRET ratio and X-Rhod fluorescence associated with Ca^2+^ release were fit using the single-exponential decay function, $y=A1^{-\left(x-x_{1}\right)\;/\tau }+y_{1}$, where *x*_*1*_ is the time in seconds of Ca^2+^ release, and *y*_*1*_ is the baseline fluorescence prior to *x*_*1*_, defined by the linear function $y=A1+y_{1}$. Differences in the characteristic time constants (τ) for each process were analyzed using a one-way ANOVA with Dunn’s post hoc test (significance = *p* < 0.05).

### Kinetic modeling

We implemented systems of ordinary differential equations according to the schematic provided in Figure 4*A* (58). This kinetic diagram describes the populations of the SERCA states under diastolic and systolic conditions. Model parameters were informed or constrained by experimental observations where appropriate, such as the PLB dissociation rates reported in this study. Mean rates were determined by averaging over several transients from multiple cells. Kinetic parameters for PLB–SERCA binding dynamics were fit to 15 independent FRET transients, while kinetic parameters for DWORF–SERCA interactions were fit to nine independent FRET measurements. All other parameters were assigned initial values that were subject to fitting against time-varying FRET data. The relative affinity of PLB for SERCA (here described by the rate fraction of *k*_on_/*k*_off_) was constrained to be twofold higher for Ca-free *versus* Ca-bound ensembles, as determined experimentally from FRET-binding curves. Likewise, the relative affinity of DWORF for SERCA (*k*_on_/*k*_off_) was constrained to increase by 25% between Ca-free and Ca-bound ensembles, consistent with FRET measurements. The ordinary differential equation system was numerically solved using the scipy (v1.5.0) SOLV_IVP function, using the experimentally measured intracellular Ca^2+^ transient as an input to the model. The resulting numerically estimated PLB–SERCA population was compared against the experimentally reported FRET data by assuming that the PLB–SERCA population was proportional to the FRET efficiency. Mean-squared error between the predicted PLB–SERCA population and the FRET data was computed to assess the fit. Fitting was optimized using a genetic algorithm we developed previously (40) that reduces the mean-squared error by randomizing model parameters and selecting those that reduce error. Forward simulations of the SERCA/PLB/DWORF equilibria for the cardiac pacing experiments used simulated Ca^2+^ transients with amplitudes and frequencies in place of those measured in HEK cells for fitting purposes. A parameter sensitivity analysis is provided in Fig. S13.

## Data availability

All code written in support of this publication are publicly available at https://github.com/huskeypm/pkh-lab-analyses.

## Supporting information

This article contains supporting information (36, 51).

## Conflict of interest

The authors declare that they have no conflicts of interest with the contents of this article.
