# Supplementary material for: Inhibitory and stimulatory micropeptides preferentially bind to different conformations of the cardiac calcium pump
Source: J Biol Chem. 2022 May 20;298(7):102060. doi: 10.1016/j.jbc.2022.102060 (PMC9218510; doi:10.1016/j.jbc.2022.102060)
Supplement: Supplemental Figures S1–S13 and Tables S1–S7 [file mmc1.pdf]

Supporting Information

Inhibitory and Stimulatory Micropeptides Preferentially Bind to Different Conformations of the Cardiac Calcium Pump

Sean R. Cleary, Xuan Fang, Ellen E. Cho, Marsha P. Pribadi, Jaroslava Seflova, Jordan R. Beach, Peter M. Kekenes-Huskey, Seth L. Robia†
Department of Cell and Molecular Physiology, Loyola University Chicago, Maywood, IL 60153

†*To Whom Correspondence Should be Addressed:* Seth L. Robia, Department of Cell and Molecular Physiology, Stritch School of Medicine, Loyola University Chicago, 2160 S. First Ave. Maywood, IL 60153
e-mail: srobia@luc.edu

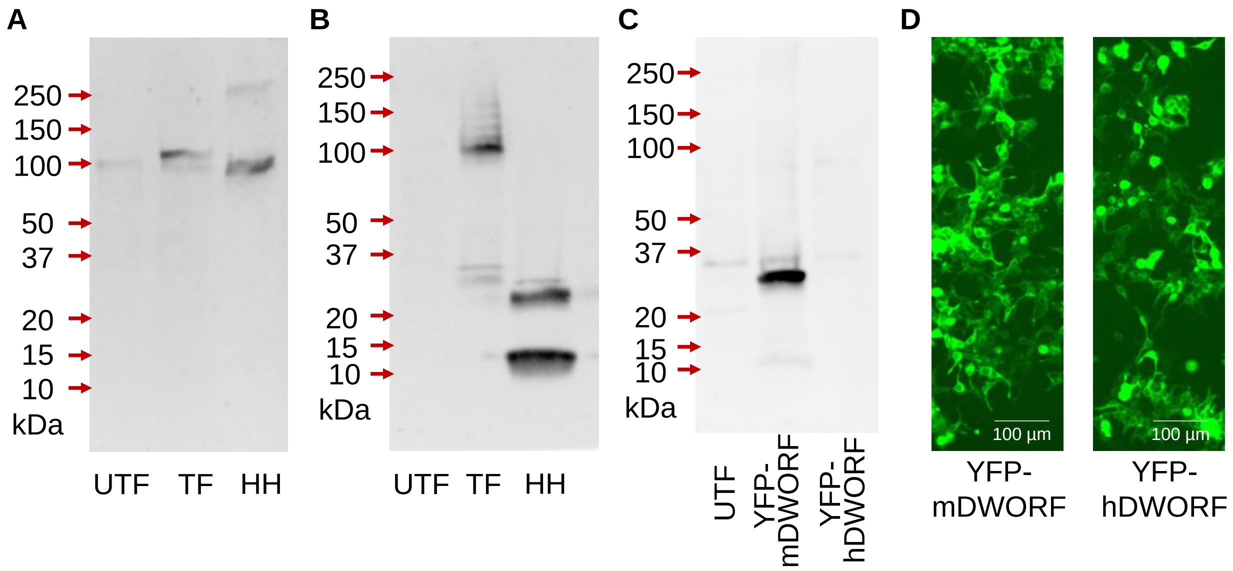

Supplementary Figure S1. Western blot analysis **A)** Anti-SERCA antibody revealed endogenous SERCA2b in untransfected cells (UTF), Cer-SERCA2a in transfected cells (TF), and SERCA2a in human heart (HH) samples. **B)** Anti-PLB antibody did not detect endogenous PLB in untransfected (UTF) cells, but showed the expected mobilities of YFP-PLB in transfected cells (TF) and PLB in the human heart (HH) sample. **C)** Anti-mouse DWORF antibody was only reactive to the mouse sequence (YFP-mDWORF), and did not bind to the human protein (YFP-hDWORF). **D)** Fluorescence microscopy showed that both mouse and human DWORF expressed well.

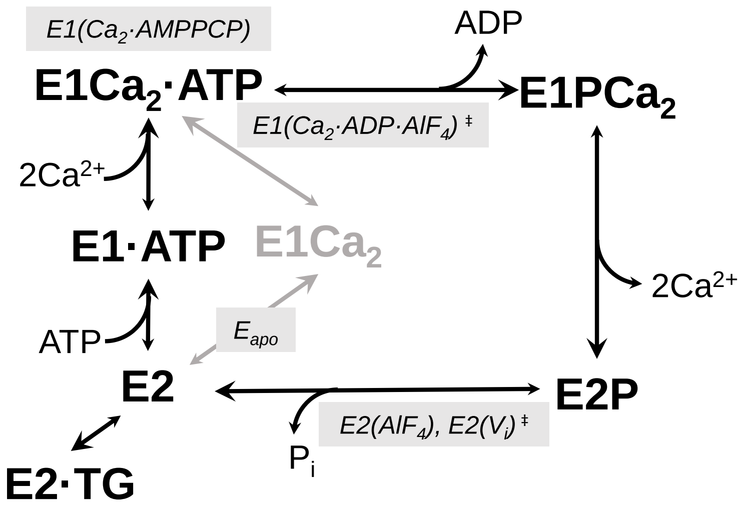

Supplementary Figure S2. Post-Albers cycle with biochemically stabilized states of SERCA. Ligand-free SERCA (pH 7) is in the E_{apo} state – a state in pH-dependent dynamic equilibrium between E2 and E1 states where substrate binding sites face the ER lumen or the cytosol respectively (1). The SERCA inhibitor, thapsigargin (TG), stabilizes a dead-end E2 conformation (1IWO) (2). Saturating concentrations of ATP or Ca^{2+} stabilize E1 conformations of the pump (3, 4). The structural analog of $E1Ca_2$-ATP where both substrates are bound is stabilized with Ca^{2+} and a non-hydrolysable ATP-analog, AMPPCP (PDB: 1T5S) (5). The ADP-bound transition state following ATP-hydrolysis is stabilized with saturating concentrations of Ca^{2+}, ADP, and the phosphate analog, AlF_4 (PDB: 2ZBD) (6). A low Ca^{2+} buffer containing either AlF_4 or the SERCA inhibitor orthovanadate (V_i) stabilize a transitional analog of the dephosphorylating E2P state (PDB: 3N5K, 5A3Q) (7, 8). ‡ indicates transition states.

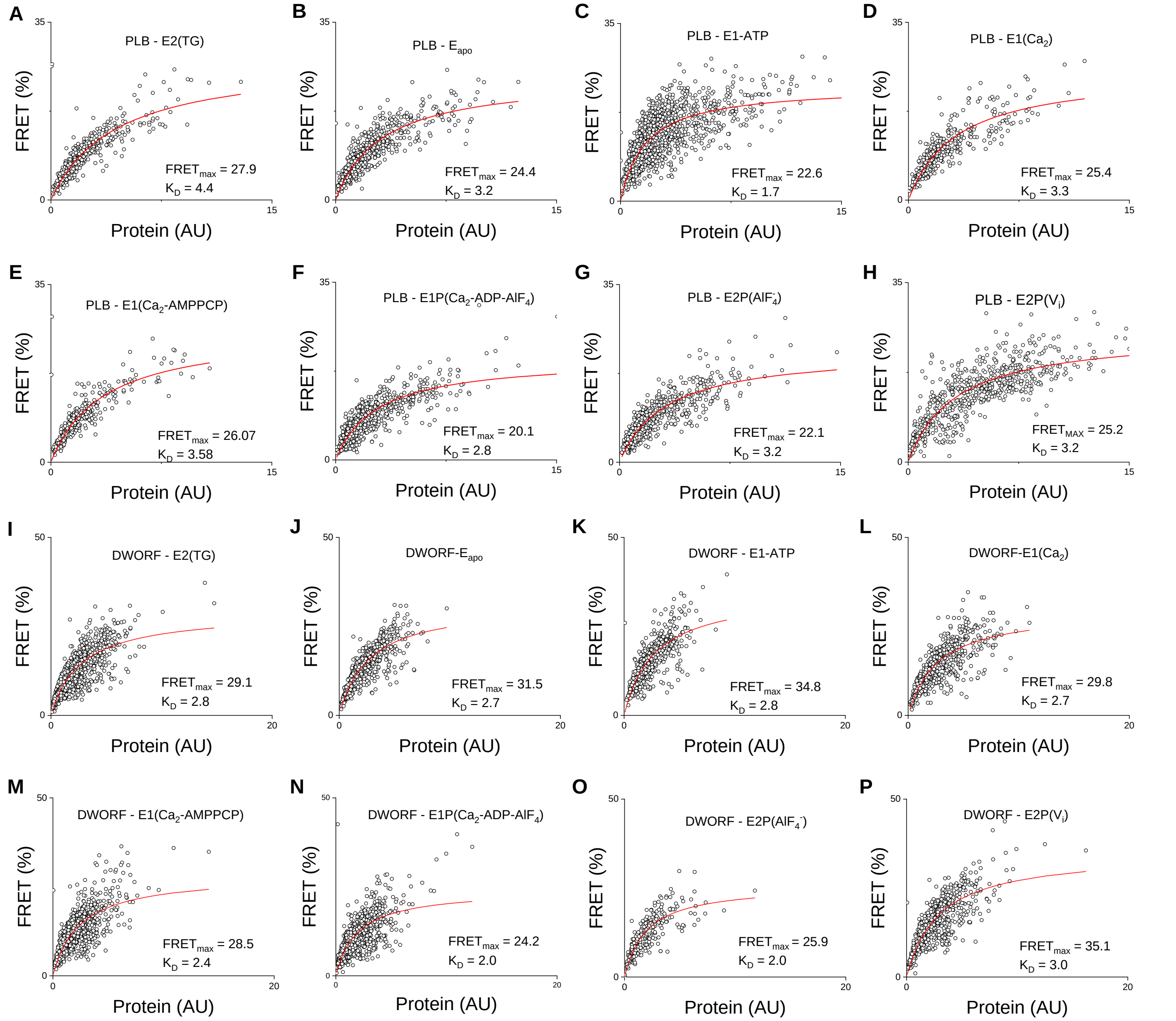

Supplementary Figure S3. Representative FRET-based binding curves for PLB and DWORF interactions with ligand-stabilized enzymatic states of SERCA. **A-H)** Representative binding curves based on FRET measurements between Cer-SERCA and YFP-PLB FRET pairs. **I-P)** Representative binding curves based on FRET measurements between Cer-SERCA and YFP-DWORF FRET pairs.

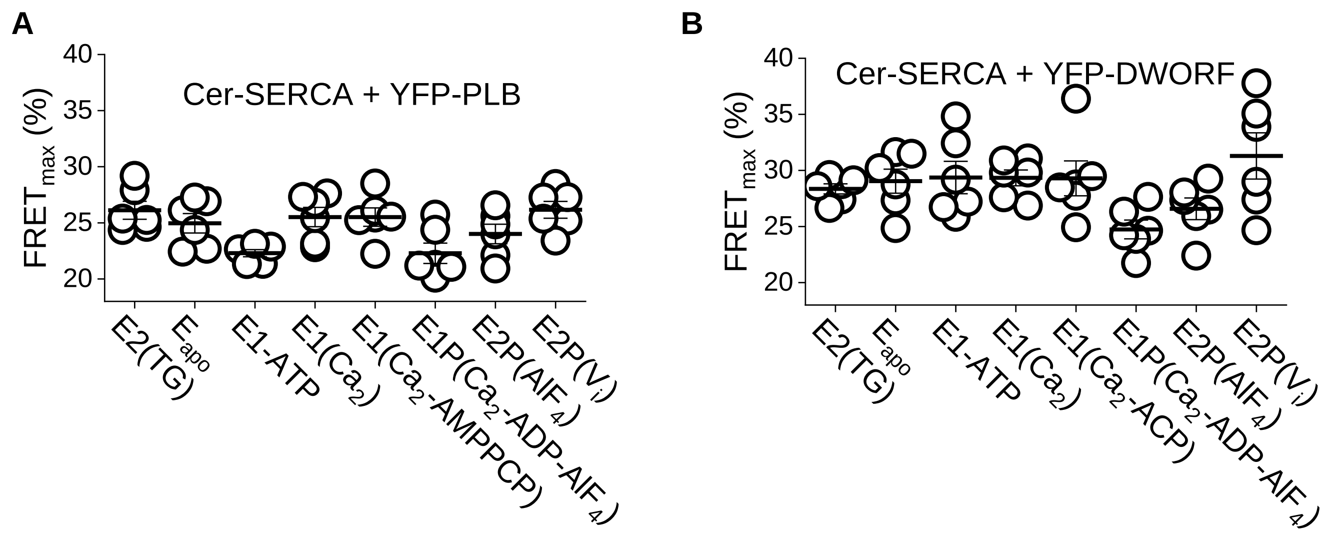

Supplementary Figure S4. FRET$_{max}$ values for PLB and DWORF regulatory complexes with ligand-stabilized enzymatic states of SERCA with lines representing mean ± SEM (n=6). Data are derived from hyperbolic fits of FRET data plotted over a range of YFP-tagged micropeptide protein concentration (See representative curves in Supplementary Fig. S2).

PLB-SERCA Conformation K_D 1-way ANOVA with Tukey's post-hoc							
	E2(TG)	E_{apo}	**E1-ATP**	E1(Ca_2)	E1(Ca_2-AMPPCP)	E1P(Ca_2-ADP-AlF_4)	E2P(AlF_4)
E2P(V_i)	5.14×10^{-2}	1	**7.07×10^{-6}***	1	1	0.67	1
E2P(AlF_4)	**1.86×10^{-2}***	1	**2.54×10^{-5}***	1	1	0.89	
E1P(Ca_2-ADP-AlF_4)	**3.93×10^{-4}***	0.97	**1.57×10^{-3}***	0.59	0.52		
E1(Ca_2-ACP)	9.13×10^{-2}	0.98	**3.33×10^{-6}***	1			
E1(Ca_2)	6.90×10^{-2}	0.99	**4.76×10^{-6}***				
**E1-ATP**	**1.96×10^{-8}***	**6.63×10^{-5}***					
E_{apo}	**8.14×10^{-3}***						

DWORF-SERCA Conformation K_D 1-way ANOVA with Tukey's post-hoc							
	E2(TG)	E_{apo}	E1-ATP	E1(Ca_2)	E1(Ca_2-AMPPCP)	E1P(Ca_2-ADP-AlF_4)	E2P(AlF_4)
E2P(V_i)	1	1	1	1	1	0.39	0.71
E2P(AlF_4)	0.73	0.95	0.95	0.88	0.86	1	
E1P(Ca_2-ADP-AlF_4)	0.41	0.73	0.73	0.59	0.56		
E1(Ca_2-ACP)	1	1	1	1			
E1(Ca_2)	1	1	1				
E1-ATP	1	1					
E_{apo}	1						

Supplementary Table S1. P values comparing differential PLB (above) and DWORF (below) dissociation constants for SERCA conformation states. Data were analyzed by 1-way ANOVA with Tukey's *post-hoc* test ($p<0.05$ = *).

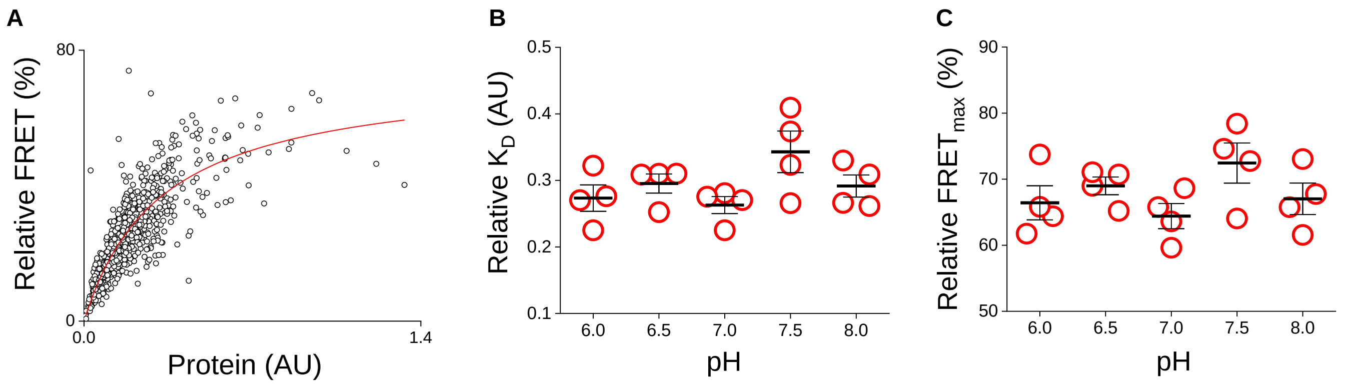

Supplementary Figure S5. PLB binding preference for E2-ATP and E1-ATP substates of the ATP bound state of SERCA was assessed by measuring Cer-SERCA – TagRFP-PLB FRET in a buffer containing ATP (3 mM) at a range of pH from 6-8 (*See Methods*). Increasingly acidic conditions protonate SERCA substrate binding sites, stabilizing E2 conformations, whereas E1 is favored at alkaline pH (1). **A)** Representative FRET-based binding curve for TagRFP-PLB interacting with ATP-bound Cer-SERCA at pH 7.0. **B)** Relative K_D values of the PLB-SERCA regulatory complex measured at a range of pH from 6-8, with lines representing mean ± SEM (n=4). We saw no change in PLB-SERCA K_D in this range, indicating no discernable preference of PLB for E2-ATP or E1-ATP states. **C)** Relative $FRET_{max}$ values of the PLB-SERCA regulatory complex measured at a range of pH from 6-8, with lines representing mean ± SEM (n=4).

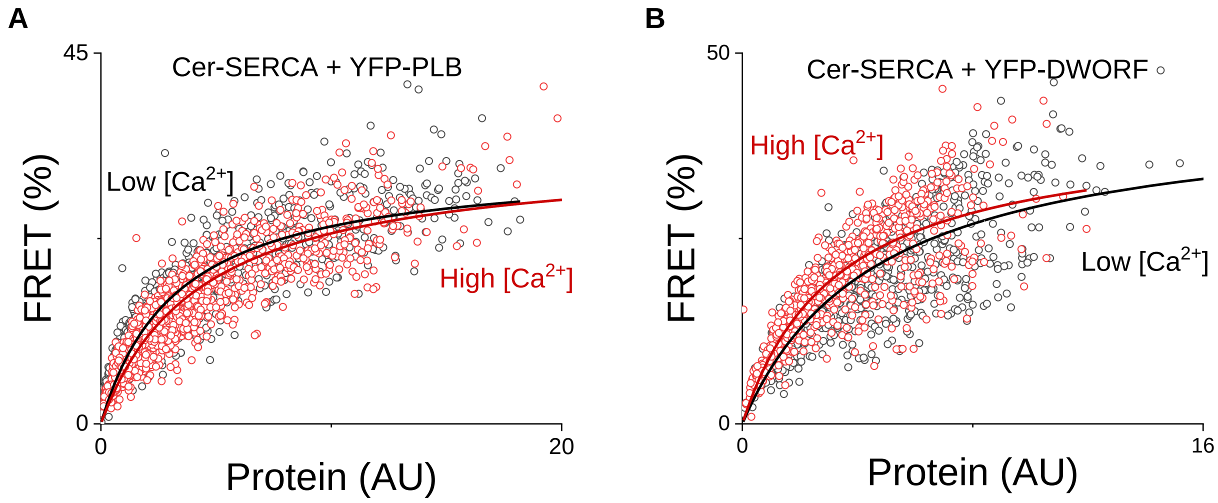

Supplementary Figure S6. Representative PLB-SERCA and DWORF-SERCA FRET-based binding curves in high and low [Ca^{2+}] solutions simulating physiological diastolic and systolic conditions in permeabilized HEK-293 cells (*See Methods*).

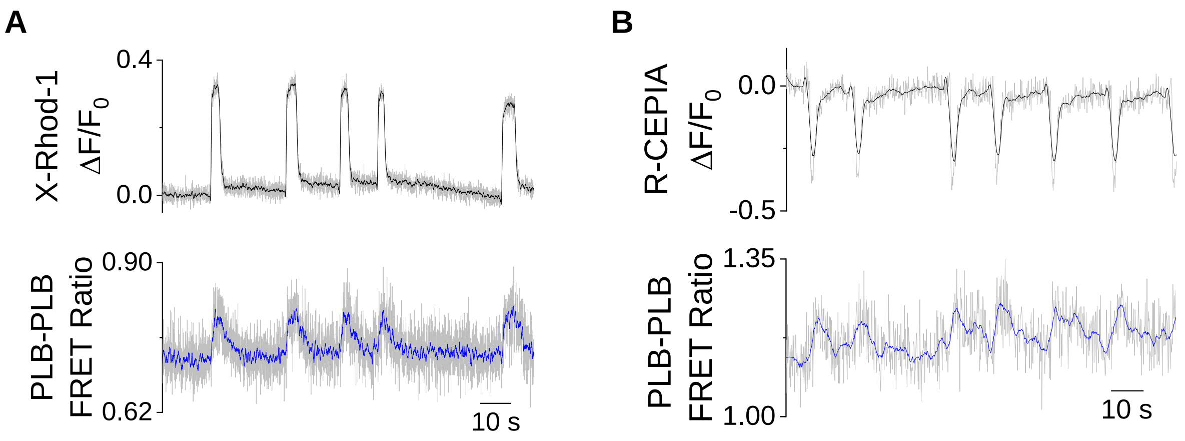

Supplementary Figure S7. Representative data for PLB - PLB FRET ratio changes measured in response to intracellular Ca^{2+} elevations. **A)** Confocal microscopy quantification of cytosolic Ca^{2+} measured by X-Rhod-1 (*grey + black smoothed trendline*) with simultaneous measurement of changes in PLB-PLB FRET (*grey + blue smoothed trendline*). **B)** Confocal microscopy quantification of ER Ca^{2+} measured by R-CEPIA (*grey + black smoothed trendline*) with simultaneous measurement of changes in PLB-PLB FRET (*grey + blue smoothed trendline*).

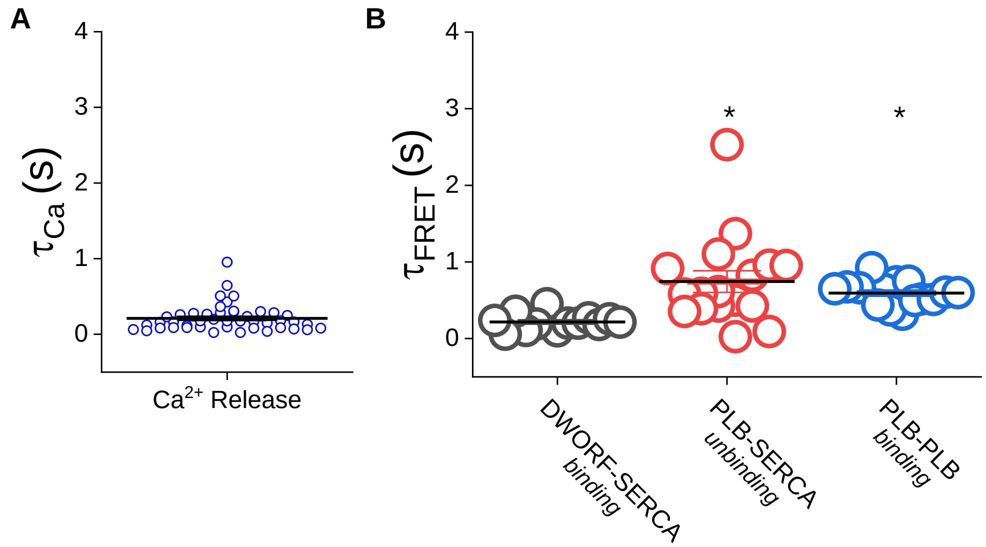

Supplementary Figure S8. PLB pentamer formation during Ca^{2+} elevations is modestly delayed by the time course of PLB-SERCA unbinding. **D)** Raw time constant (τ) values for Ca^{2+} release derived from single exponential decay fits of increases in X-rhod-1 fluorescence. **E)** Raw time constant (τ) values for SERCA and micropeptide binding processes derived from single exponential decay fits of FRET ratio changes associated with Ca^{2+} release with lines representing mean ± SEM. Differences determined by one-way ANOVA with Dunn's *post-hoc* test (* = $p<0.05$, see Table S3 for complete statistical analysis). Data are shown in Fig. 3E as the FRET latency quantified by the difference between the τ of FRET ratio changes and the τ their corresponding Ca^{2+} uptake event.

Apparent τ (Mean ± SEM)	
Process	τ (s)
DWORF – SERCA (*binding)*	0.22 ± 0.03
PLB – SERCA (*unbinding)*	0.74 ± 0.14
PLB – PLB (*binding)*	0.59 ± 0.04
Ca^{2+} Release	0.21 ± 0.03

Supplementary Table S2. Time constants (τ) quantified for regulatory binding processes associated with Ca^{2+} release. Time constant values are reported as mean ± SEM.

SERCA/micropeptide binding/unbinding processes analyzed by 1-way ANOVA with Dunn's post-hoc	DWORF-SERCA (*binding*)	PLB-SERCA (*unbinding*)	PLB-PLB (*binding*)
Ca^{2+} Release	1	**3.84 x 10^{-8}***	**1.74 x 10^{-4}***
PLB-PLB (*binding*)	**5.79 x 10^{-3}***	0.62	
PLB-SERCA (*unbinding*)	**2.45 x 10^{-5}***		

Supplementary Table S3. P values comparing differences in time constants (τ) for SERCA/micropeptide binding/unbinding processes analyzed by 1-way ANOVA with Dunn's *post-hoc* test. See Fig. S7 and Table S2 for summary data.

FRET Latency Δτ ($\tau_{FRET} - \tau_{Ca\ Release}$) 1-way ANOVA with Dunn's post-hoc test	PLB-SERCA (*unbinding*)	PLB-PLB (*binding*)
DWORF-SERCA (*binding*)	**4.06 x 10^{-3}***	**1.08 x 10^{-3}***
PLB-PLB (*binding*)	0.92	

Supplementary Table S4. P values comparing FRET latency Δτ (τ_{FRET} -$\tau_{Ca\ Release}$) differences between groups analyzed by 1-way ANOVA with Dunn's *post-hoc* test. See Fig. 3E for summary data.

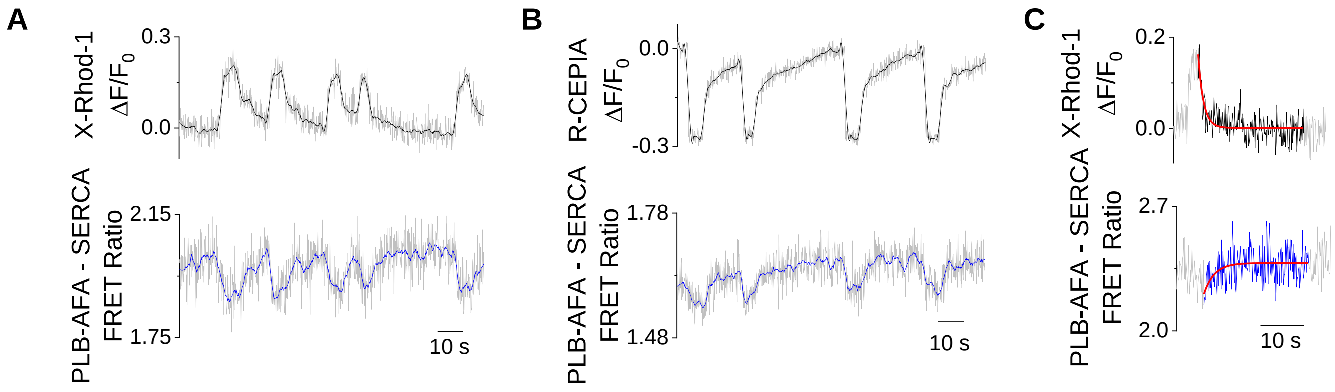

Supplementary Figure S9. Representative data for PLB-AFA - SERCA FRET ratio changes measured in response to intracellular Ca^{2+} elevations. **A)** Confocal microscopy quantification of cytosolic Ca^{2+} measured by X-Rhod-1 (*grey + black smoothed trendline*) with simultaneous measurement of changes in PLB-AFA - SERCA FRET (*grey + blue smoothed trendline*). **B)** Confocal microscopy quantification of ER Ca^{2+} measured by R-CEPIA (*grey + black smoothed trendline*) with simultaneous measurement of changes in PLB-AFA - SERCA FRET (*grey + blue smoothed trendline*). **C)** Representative single exponential decay fit of PLB-AFA – SERCA rebinding during Ca^{2+} uptake.

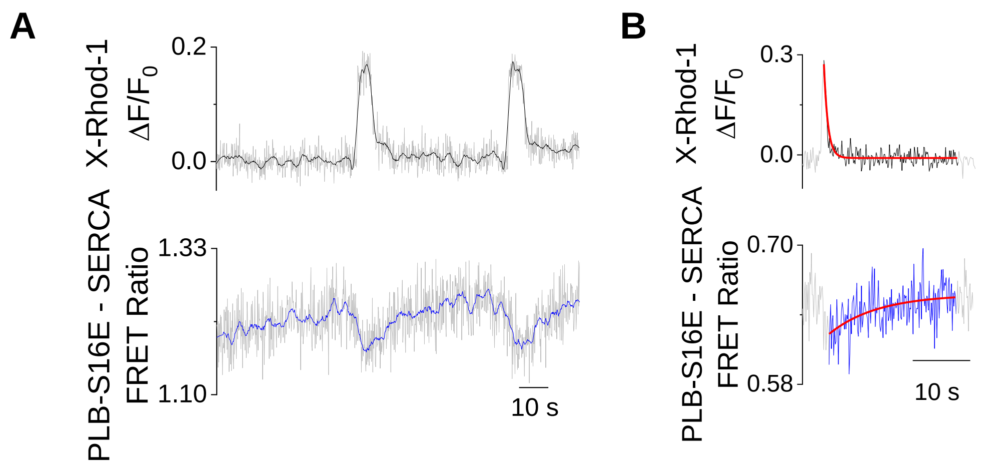

Supplementary Figure S10. Representative data for PLB-S16E - SERCA FRET ratio changes measured in response to intracellular Ca^{2+} elevations. **A)** Confocal microscopy quantification of cytosolic Ca^{2+} measured by X-Rhod-1 (*grey + black smoothed trendline*) with simultaneous measurement of changes in PLB-S16E - SERCA FRET (*grey + blue smoothed trendline*). **B)** Representative single exponential decay fit of PLB-S16E – SERCA rebinding during Ca^{2+} uptake.

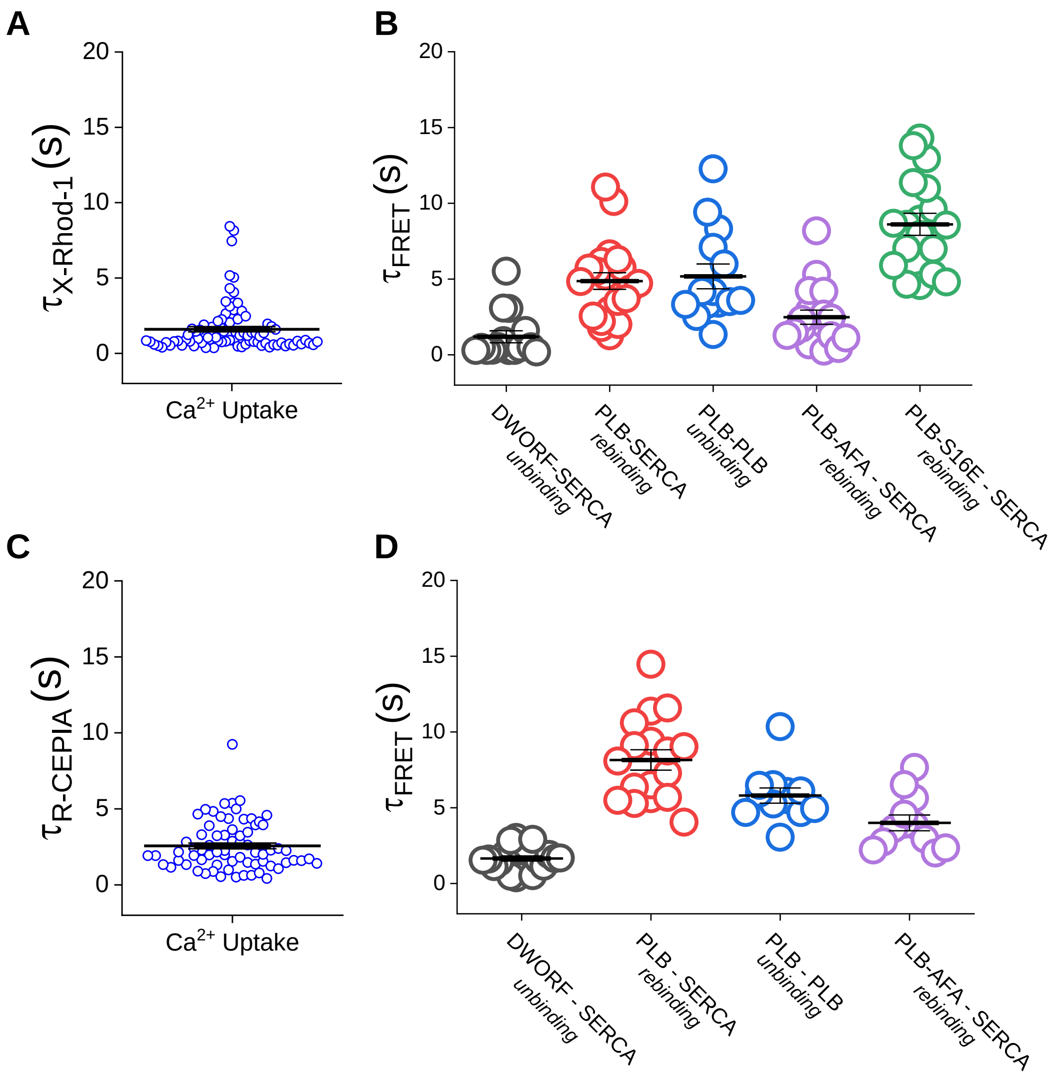

Supplementary Figure S11. Time constants (τ) quantified for regulatory binding processes associated with Ca^{2+} uptake with lines representing mean ± SEM. **A)** Time constant values for Ca^{2+} uptake derived from single exponential decay fits of decreases in X-rhod-1 fluorescence. **B)** Raw time constant values for SERCA and micropeptide binding processes derived from single exponential decay fits of FRET ratio changes associated with Ca^{2+} uptake measured by X-Rhod-1 fluorescence. Data are shown in Fig. 3J as the FRET latency quantified by the difference between the τ of FRET ratio changes and the τ their corresponding Ca^{2+} uptake event. See Table S6 for complete statistical analysis. **C)** Time constant values for Ca^{2+} uptake derived from single exponential decay fits of increases in R-CEPIA1er fluorescence. **D)** Raw time constant values for SERCA and micropeptide binding processes derived from single exponential decay fits of FRET ratio changes associated with Ca^{2+} uptake measured by R-CEPIA1er fluorescence.

Apparent τ (Mean ± SEM)	
Process	τ (s)
DWORF – SERCA (*unbinding*)	1.2 ± 0.4
PLB – SERCA (*rebinding*)	4.9 ± 0.6
PLB – PLB (*unbinding*)	5.2 ± 0.8
PLB-AFA – SERCA *(rebinding)*	2.5 ± 0.5
PLB-S16E – SERCA *(rebinding)*	8.6 ± 0.7
Ca^{2+} Uptake	1.6 ± 0.2

Supplementary Table S5. Time constants (τ) of binding processes associated with Ca^{2+} uptake measured by X-Rhod-1 fluorescence. Time constant values are reported as mean ± SEM.

SERCA/Micropeptide binding/unbinding τ 1-way ANOVA with Dunn's post-hoc	DWORF-SERCA (*unbinding*)	PLB-SERCA (*rebinding*)	PLB-PLB (*unbinding*)	PLB-S16E - SERCA (*rebinding*)	PLB-AFA – SERCA (*rebinding*)
Ca^{2+} Uptake	1	**2.38 x 10^{-8}***	**2.81 x 10^{-7}***	**<1 x 10^{-10}***	0.81
PLB-AFA - SERCA (*rebinding*)	0.72	**7.95 x 10^{-3}***	**6.30 x 10^{-3}***	**<1 x 10^{-10}***	
PLB-S16E - SERCA (*rebinding*)	**<1 x 10^{-10}***	**1.64 x 10^{-6}***	**1.30 x 10^{-4}***		
PLB - PLB (*unbinding*)	**1.35 x 10^{-5}***	1			
PLB - SERCA (*rebinding*)	**9.79 x 10^{-6}***				

Supplementary Table S6. P values comparing differences in time constants (τ) for SERCA-micropeptide binding/unbinding processes analyzed by 1-way ANOVA with Dunn's *post-hoc* test. See Fig. S10 and Table S5 for summary data.

FRET Latency Δτ ($\tau_{FRET} - \tau_{Ca\ Uptake}$) 1-way ANOVA with Dunn's post-hoc test	DWORF-SERCA (*unbinding*)	PLB-SERCA (*rebinding*)	PLB-PLB (*unbinding*)	PLB-S16E - SERCA (*rebinding*)
PLB-AFA - SERCA (*rebinding*)	0.85	**2.24 x 10^{-2}***	**1.73 x 10^{-3}***	**3.48 x 10^{-8}***
PLB-S16E - SERCA (*rebinding*)	**2.96 x 10^{-10}***	**3.59 x 10^{-3}***	0.23	
PLB-PLB (*unbinding*)	**2.45 x 10^{-5}***	0.95		
PLB-SERCA (*rebinding*)	**3.03 x 10^{-4}***			

Supplementary Table S7. P values comparing FRET latency Δτ (τ_{FRET} -$\tau_{Ca\ Uptake}$) differences between groups analyzed by 1-way ANOVA with Dunn's *post-hoc* test. See Fig. 3J for summary data.

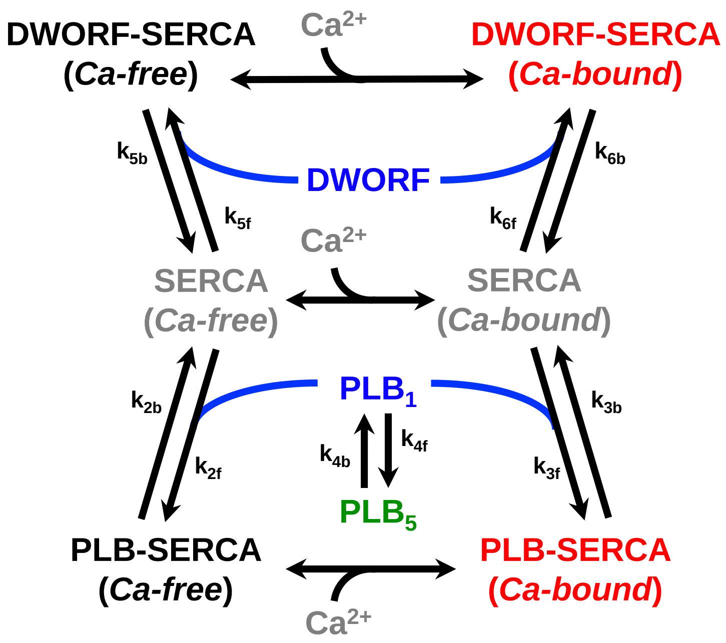

Parameter	Value (s^{-1})	SEM (s^{-1})
k_{2f}	8.1×10^3	5.8×10^2
k_{2b}	1.1×10^2	3.3×10^0
k_{3f}	5.6×10^3	6.7×10^2
k_{3b}	1.7×10^2	3.2×10^1
k_{4f}	1.0×10^1	9.0×10^{-1}
k_{4b}	7.9×10^{-2}	1.8×10^{-2}
k_{5f}	3.5×10^1	7.5×10^0
k_{5b}	1.9×10^1	8.5×10^0
k_{6f}	1.3×10^4	1.4×10^3
k_{6b}	4.8×10^3	1.8×10^3

Supplementary Figure S12. A reaction diagram indicating the kinetic parameters derived from experimental measurements. Mean kinetic parameters of PLB -SERCA and -pentamer (PLB_5) binding dynamics were fit from PLB-SERCA FRET experiments, and mean kinetic parameters of DWORF-SERCA binding dynamics were fit from DWORF-SERCA FRET experiments (*see Methods*).

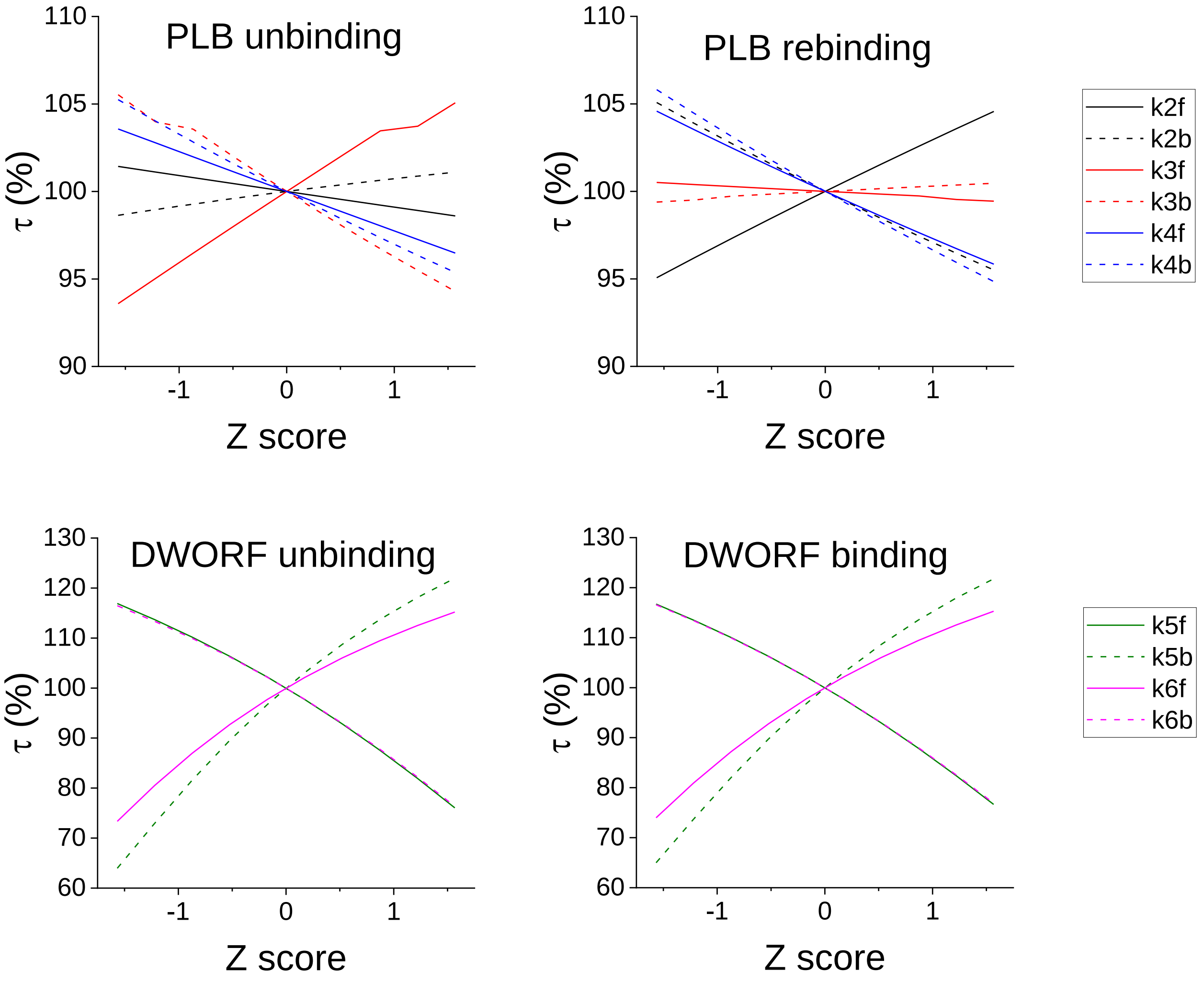

Supplementary Figure S13. Parameter sensitivity analysis describing how simulated PLB-SERCA and DWORF-SERCA binding dynamics are sensitive to changes in experimentally obtained parameters.

Supplementary Methods

To simulate the Ca^{2+} transient, we used the following equations:

$$\mathrm{Ca(t)} = \begin{cases} 0 & t < t_{init} \\ \frac{(t-t_{init})^n}{K+(t-t_{init})^n} & t_{init} < t < t_{init} + t_{setpoint} \\ \frac{K}{K+(t-t_{init})^n} & t > t_{init} + t_{setpoint} \end{cases} \quad \text{eq. S1}$$

where t_{init} is the time duration of basal Ca^{2+}, $t_{setpoint}$ is the time duration of peak Ca^{2+}, and K and n are Ca^{2+} transient shape parameters.

As a simplification, we assume that SERCA exists in two ensembles: diastolic and systolic. Furthermore, these two ensembles distribute following the Ca^{2+} transient, which can be described by the following equations:

$$SERCA_{dia,tot} = SERCA_{tot} * \frac{Ca(t)}{Ca_{max}} \quad \text{eq. S2}$$

$$SERCA_{sys,tot} = SERCA_{tot} - SERCA_{sys} \quad \text{eq. S3}$$

where $SERCA_{dia,tot}$ is the diastolic SERCA ensemble, $SERCA_{sys,tot}$ is the systolic SERCA ensemble, and $SERCA_{tot}$ is the total amount of SERCA.

The following ordinary differential equations were used to describe the models of PLB-SERCA and DWORF-SERCA binding.

PLB-SERCA

$$\frac{dPLB\cdot SERCA_{dia}}{dt} = k_{2f} * PLB_1 * SERCA_{dia} - k_{2b} * PLB \cdot SERCA_{dia} \quad \text{eq. S4}$$

$$\frac{dPLB\cdot SERCA_{sys}}{dt} = k_{3f} * PLB_1 * SERCA_{sys} - k_{3b} * PLB \cdot SERCA_{sys} \quad \text{eq. S5}$$

$$\frac{dPLB_5}{dt} = k_{4f} * PLB_1 - k_{4b} * PLB_5 \quad \text{eq. S6}$$

DWF-SERCA

$$\frac{dDWORF\cdot SERCA_{dia}}{dt} = k_{5f} * DWORF * SERCA_{dia} - k_{5b} * DWORF \cdot SERCA_{dia} \quad \text{eq. S7}$$

$$\frac{dDWORF\cdot SERCA_{sys}}{dt} = k_{6f} * DWORF * SERCA_{sys} - k_{6b} * DWORF \cdot SERCA_{sys} \quad \text{eq. S8}$$

where $SERCA_{dia}$ and $SERCA_{sys}$ are free diastolic and systolic SERCA, $PLB\cdot SERCA_{dia}$ and $PLB\cdot SERCA_{sys}$ are diastolic and systolic PLB-SERCA complexes, $DWORF\cdot SERCA_{dia}$ and $DWORF\cdot SERCA_{sys}$ are diastolic and systolic DWORF-SERCA complexes, PLB_1 is monomeric PLB, PLB_5 is pentameric PLB, and *DWORF* is free DWORF.

Supplementary Information References:

1. Hughes G, Starling AP, Sharma RP, East JM, Lee AG. An investigation of the mechanism of inhibition of the Ca(2+)-ATPase by phospholamban. Biochem J. 1996;318 (Pt 3):973-9. Epub 1996/09/15. doi: 10.1042/bj3180973. PubMed PMID: 8836146; PMCID: PMC1217713.
2. Toyoshima C, Nomura H. Structural changes in the calcium pump accompanying the dissociation of calcium. Nature. 2002;418(6898):605-11. Epub 2002/08/09. doi: 10.1038/nature00944. PubMed PMID: 12167852.
3. Raguimova ON, Smolin N, Bovo E, Bhayani S, Autry JM, Zima AV, Robia SL. Redistribution of SERCA calcium pump conformers during intracellular calcium signaling. Journal of Biological Chemistry. 2018;293(28):10843-56. doi: 10.1074/jbc.RA118.002472.
4. Toyoshima C, Nakasako M, Nomura H, Ogawa H. Crystal structure of the calcium pump of sarcoplasmic reticulum at 2.6 A resolution. Nature. 2000;405(6787):647-55. Epub 2000/06/23. doi: 10.1038/35015017. PubMed PMID: 10864315.
5. Sorensen TL, Moller JV, Nissen P. Phosphoryl transfer and calcium ion occlusion in the calcium pump. Science. 2004;304(5677):1672-5. Epub 2004/06/12. doi: 10.1126/science.1099366. PubMed PMID: 15192230.
6. Toyoshima C, Nomura H, Tsuda T. Lumenal gating mechanism revealed in calcium pump crystal structures with phosphate analogues. Nature. 2004;432(7015):361-8. Epub 2004/09/28. doi: 10.1038/nature02981. PubMed PMID: 15448704.
7. Clausen JD, Bublitz M, Arnou B, Olesen C, Andersen JP, Moller JV, Nissen P. Crystal Structure of the Vanadate-Inhibited Ca(2+)-ATPase. Structure. 2016;24(4):617-23. Epub 2016/04/07. doi: 10.1016/j.str.2016.02.018. PubMed PMID: 27050689.
8. Bublitz M, Musgaard M, Poulsen H, Thogersen L, Olesen C, Schiott B, Morth JP, Moller JV, Nissen P. Ion pathways in the sarcoplasmic reticulum Ca2+-ATPase. J Biol Chem. 2013;288(15):10759-65. Epub 2013/02/13. doi: 10.1074/jbc.R112.436550. PubMed PMID: 23400778; PMCID: PMC3624456.
